# Phase Diagrams and Piezoelectric Properties of Wurtzite Al_1−*x*−*y*_Sc_*x*_Gd_*y*_N Heterostructural Alloys

**DOI:** 10.1002/advs.202520641

**Published:** 2026-01-30

**Authors:** Julia L. Martin, Cheng‐Wei Lee, Nate S.P. Bernstein, Thi Nguyen, Ande Bryan, Eli Cooper, John S. Mangum, Sage R. Bauers, Andriy Zakutayev, Keisuke Yazawa, Prashun Gorai, Rebecca W. Smaha

**Affiliations:** ^1^ National Renewable Energy Laboratory Golden Colorado USA; ^2^ Colorado School of Mines Golden Colorado USA; ^3^ Rensselaer Polytechnic Institute Troy New York USA

**Keywords:** DFT calculations, ferroelectric, nitrides, sputtering, wurtzites

## Abstract

Ternary nitride alloys based on wurtzite AlN are a promising platform to realize functional materials, particularly ferroelectrics and optical emitters, that can smoothly integrate with conventional microelectronics. Here, a strategic design is presented to enable multifunctional materials by substituting multiple elements into AlN to create quaternary nitride alloys. By combining computational predictions and combinatorial thin film synthesis, the phase diagram of these quaternary Al–Sc–Gd–N alloys (or pseudo‐ternary heterostructural AlN–ScN–GdN alloys) is successfully predicted as a function of effective temperature, and we experimentally grow ${\mathrm{Al}}_{1-x-y}{\mathrm{Sc}}_{x}{\mathrm{Gd}}_{y}N$ thin films for the first time. It is revealed that ${\mathrm{Al}}_{1-x-y}{\mathrm{Sc}}_{x}{\mathrm{Gd}}_{y}N$ crystallizes in a wurtzite‐derived structure for x+y≲0.35, consistent with the calculated phase diagram. The computational investigation explores whether co‐substitution induces cooperative effects on these alloys' piezoelectric and ferroelectric properties, finding that it is beneficial for reducing the polarization switching barrier. We calculate that ${\mathrm{Al}}_{1-x-y}{\mathrm{Sc}}_{x}{\mathrm{Gd}}_{y}N$ thin films should display ferroelectric switching. This is supported by our experimental measurements of a high optical bandgap, enhanced piezoelectric coefficient, and a change in the calculated polarization switching mechanism, and we achieve preliminary ferroelectric switching that experimentally realizes the prediction. Overall, our work sets the foundation toward quaternary wurtzite‐nitride‐based multifunctional materials, including piezoelectrics, ferroelectrics, and possibly even multiferroics.

## Graphical abstract

This study demonstrates ferroelectricity and piezoelectric properties improvement of quaternary wurtzite ${\mathrm{Al}}_{1-x-y}{\mathrm{Sc}}_{x}{\mathrm{Gd}}_{y}N$ films, guided by density functional theory calculations. Wurtzite ${\mathrm{Al}}_{1-x-y}{\mathrm{Sc}}_{x}{\mathrm{Gd}}_{y}N$ films have a high optical bandgap, enhanced piezoelectric coefficient, and ferroelectricity as predicted by the calculated polarization switching mechanism. Our work sets the foundation toward quaternary wurtzite‐nitride‐based multifunctional materials, including piezoelectrics, ferroelectrics, and possibly even multiferroics.

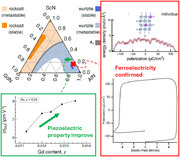

## 1 Introduction

Among emerging functional materials, wurtzite‐structured nitrides, such as aluminum nitride (AlN) and gallium nitride (GaN), are particularly attractive for their inherently polar crystal structure, robust electric dipoles, and structural compatibility with conventional substrates such as Si, ${\mathrm{Al}}_{2}O_{3}$, and GaN. Among wurtzite‐structured ternary nitrides, AlN‐based heterostructural alloys have already shown promise as electromechanical resonators [1] and ferroelectric materials [2, 3, 4, 5, 6]. Furthermore, wurtzite‐structured III‐nitrides are amenable to tunability of their electronic properties via chemistry, strain, and film thickness, which is strongly desirable for the aforementioned applications. In light of such properties as well as recent reports demonstrating strong ferroelectricity in ${\mathrm{Al}}_{1-x}{\mathrm{Sc}}_{x}N$ [5, 7, 8] and ${\mathrm{Al}}_{1-x}{\mathrm{Gd}}_{x}N$, [9] we consider this materials class a strong candidate platform for realizing new multifunctional materials.

While ternary nitrides derived from AlN are now common, quaternary nitrides are the next frontier in materials design within this family: they expand the range of possible alloy engineering for piezoelectricity and ferroelectricity in wurtzite nitride alloys, and incorporating multiple elements could induce many different functionalities simultaneously. To date, there have been scant experimental reports of quaternaries, and most involve co‐substitution of Sc with another trivalent cation. Er and ${\mathrm{Ni}}^{4+}$ have been incorporated at very low levels [10, 11], while higher levels of B substitution (y≈ 6–16% in ${\mathrm{Al}}_{1-x-y}{\mathrm{Sc}}_{x}B_{y}N$) were shown to improve leakage current but have a complex effect on other ferroelectric properties [12]. Computation of substituting high levels of ${\mathrm{Hf}}^{4+}$ or ${\mathrm{Zr}}^{4+}$ into ${\mathrm{Al}}_{1-x}{\mathrm{Sc}}_{x}N$ predicted strong piezoelectric properties [13].

Nonetheless, synthesis of ${\mathrm{Al}}_{1-x}M_{x}N$ alloys is difficult; they are highly metastable because many metal nitrides are stable in the rocksalt structure instead of the wurtzite structure, creating a high synthetic activation barrier. Incorporating enough Sc to induce desirable ferroelectric properties has been the subject of intense study, and we showed previously that the effective synthetic temperature for ${\mathrm{Al}}_{1-x}RE_{x}N$ (*RE* = Gd, Tb, Pr) is on the order of ∼ 3000 K [14, 15]. Because of this, highly non‐equilibrium synthetic conditions are required. In addition to this fundamental thermodynamic challenge, there is a significant increase in synthetic variables moving from ternary to quaternary alloys, which is a further experimental undertaking.

Among candidate elements to substitute for Al, rare earth cations are particularly attractive as they may impart optoelectronic and/or magnetic functionality. As such, we hypothesize that substitution of wurtzite‐structured, AlN‐based III‐nitrides with trivalent rare earth cations such as ${\mathrm{Gd}}^{3+}$, ${\mathrm{Nd}}^{3+}$, or ${\mathrm{Tb}}^{3+}$ may induce some degree of simultaneous magnetic and ferroelectric ordering. This would be of high interest as a potential single‐phase multiferroic material, which are a promising materials solution to improve the efficiency of computing and data storage due to their unique properties: the simultaneous ordering of electric and magnetic dipoles enables fast and energy‐efficient electrical data writing and non‐destructive magnetic data reading [16]. This modality will increase the versatility of wurtzite (multi)ferroic materials into memory devices beyond the conventional memory architectures such as ferroelectric random access memory and ferroelectric field effect transistors suggested in literature [17, 18]. Conventional multiferroic materials are based mostly on oxide perovskites and hexagonal rare‐earth manganites and tend to suffer from oxidative instability, oxygen vacancies, and integration challenges into device‐relevant semiconductor platforms such as Si, SiC, and GaN, which collectively limit widespread application [19, 20, 21]. In addition, multiferroicity at room temperature in single phase materials has only been demonstrated in very few material systems and all of them face challenges considering heterogeneous integration.

However, many fundamental questions must be investigated about this emerging family of quaternary AlN‐based materials before they can be realized in applications. Are they thermodynamically stable, and if so at what composition ranges? What is the stable crystal structure at each composition? What conditions are necessary to synthesize them? What is the effect of co‐substitution upon properties such as bandgap, piezoelectric coefficient, and ferroelectric switching? Are there any cooperative effects resulting from co‐substitution?

Given our success in separately modeling and synthesizing wurtzite‐structured ${\mathrm{Al}}_{1-x}{\mathrm{Sc}}_{x}N$ [5, 6, 22] and ${\mathrm{Al}}_{1-x}{\mathrm{RE}}_{x}N$ (RE = Gd, Tb, Pr) [14, 15, 23], we were motivated to explore co‐alloying AlN with ScN and GdN to yield quaternary ${\mathrm{Al}}_{1-x-y}{\mathrm{Sc}}_{x}{\mathrm{Gd}}_{y}N$. Herein, we demonstrate the first synthesis of quaternary ${\mathrm{Al}}_{1-x-y}{\mathrm{Sc}}_{x}{\mathrm{Gd}}_{y}N$ in a wurtzite‐derived crystal structure with Sc content of *x* ≈0.20 or *x* ≈0.30 and Gd incorporation up to *y* ≲ 0.15. We present the results of our computationally‐guided experimental investigation into the heterostructural co‐alloying of wurtzite AlN, rocksalt ScN, and rocksalt GdN with a view toward multiferroic applications. We employed density functional theory (DFT) calculations to predict phase diagrams from 0 to 4000 K for pseudo‐ternary AlN–ScN–GdN alloys. For clarity, these will henceforth be referred to as quaternary AlN–ScN–GdN alloys. Motivated by promising cooperative effects arising from co‐substitution of Sc and Gd, we support these predictions experimentally by depositing combinatorial films of ${\mathrm{Al}}_{1-x-y}{\mathrm{Sc}}_{x}{\mathrm{Gd}}_{y}N$ with radio‐frequency magnetron co‐sputtering and performing structural and compositional characterization as well as optical, piezoelectric, and preliminary ferroelectric switching measurements. In concert, X‐ray diffraction and X‐ray fluorescence support the first successful synthesis of this emerging quaternary AlN‐based material in a wurtzite‐derived crystal structure, and our functional properties testing supports ${\mathrm{Al}}_{1-x-y}{\mathrm{Sc}}_{x}{\mathrm{Gd}}_{y}N$ as a candidate piezoelectric material.

## 2 Results and Discussion

### 2.1 Prediction of Quaternary ${\mathrm{Al}}_{1-x-y}{\mathrm{Sc}}_{x}{\mathrm{Gd}}_{y}N$ Alloy Stability

AlN‐based heterostructural alloys have potential for multifunctional properties, specifically the enhancement of piezoelectric properties and the coexistence of ferroelectricity and magnetic order or optical emission; however, their synthesis is highly non‐trivial. To guide the synthesis, we have developed a computational workflow to predict the thermodynamics that underpin AlN–ScN–GdN quaternary alloys. Building upon our previous work on pseudo‐binary (i.e., ternary) alloys of AlN‐ScN and AlN‐*RE*N (where *RE* = rare earth elements) [5, 9, 14, 22, 24], and inspired by the methodologies established for multi‐component metal alloys [25, 26], we generalize our previous workflow and predict quaternary phase diagrams for a range of effective temperatures.

**FIGURE 1:**
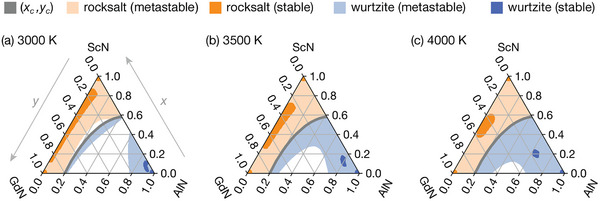
Calculated phase diagrams for ${\mathrm{Al}}_{1-x-y}{\mathrm{Sc}}_{x}{\mathrm{Gd}}_{y}N$ alloys across a range of effective temperatures (3000 – 4000 K). The unstable regions are unshaded, and the metastable and stable regions are shaded. The vertices represent stable wurtzite AlN, rocksalt ScN, and rocksalt GdN. The stability region of the binary end‐member compounds is magnified for clarity.

Figure 1 presents the calculated AlN–ScN–GdN phase diagrams for a temperature range of 3000 to 4000 K. Figure 1 is a standard alloy phase diagram illustrating regions of thermodynamic stability, metastability, and instability across composition and temperature. The diagrams distinguish between regions of alloy instability (unshaded regions, corresponding to spinodal decomposition), metastability (lightly shaded regions), and thermodynamic stability (dark shaded regions). These alloys are inherently heterostructural because they involve alloying wurtzite AlN with rocksalt ScN and GdN. As such, a compositional boundary emerges, defined by a critical composition line ($x_{c}$, $y_{c}$), beyond which the alloyed material transitions from the wurtzite to rocksalt phase. Although this phase boundary is diffuse in practice, it consistently appears in the phase diagrams as a temperature‐independent demarcation separating the wurtzite‐rich region near the AlN vertex from the rocksalt‐dominant region.

A natural question arises as to why we consider such high temperatures that are well beyond typical experimental synthesis conditions. These temperatures are effective temperatures (not actual temperatures) that provide a conceptual link between equilibrium thermodynamic predictions and non‐equilibrium synthesis processes such as reactive sputtering, which was utilized here. The formation of metastable phases during such growth techniques is governed by multiple factors, including but not limited to substrate temperature, flux bombardment energies, kinetic barriers, and energy dissipation pathways. As such, we adopt the notion of an emergent effective temperature, $T_{eff}$, which serves as a unifying parameter that captures the average thermodynamic driving forces under far‐from‐equilibrium conditions. While $T_{eff}$ cannot be precisely defined or controlled, its approximate value may be inferred from successful synthesis of a given alloy composition. These insights may then be transferred to neighboring compositions, assuming similar growth conditions and kinetic constraints, positing the computed phase diagrams as a predictive framework for guiding the synthesis of ${\mathrm{Al}}_{1-x-y}{\mathrm{Sc}}_{x}{\mathrm{Gd}}_{y}N$ alloys under non‐equilibrium conditions.

Our prior work on the closely related ${\mathrm{Al}}_{1-x}{\mathrm{Gd}}_{x}N$ alloy revealed that effective temperatures of approximately 3000 and 4000 K are required to (meta)stabilize compositions with x > 0.2 and x > 0.3, respectively. [14] These predictions were borne out experimentally: the successful synthesis of single‐phase wurtzite ${\mathrm{Al}}_{1-x}{\mathrm{Gd}}_{x}N$ thin films with x up to 0.25 by reactive sputtering suggests that the effective temperature achieved during this non‐equilibrium growth falls within the 3000–4000 K range [14]. This provides a crucial benchmark for estimating $T_{eff}$ in related systems. By extension, we assume that similar $T_{eff}$ can be accessed during the co‐alloying of AlN with both Sc and Gd via comparable reactive sputtering techniques. Therefore, the phase diagrams most relevant to thin‐film growth of ${\mathrm{Al}}_{1-x-y}{\mathrm{Sc}}_{x}{\mathrm{Gd}}_{y}N$ are those calculated at 3000, 3500, and 4000 K (Figure 1a–c). These phase diagrams predict metastable wurtzite regions that expand in size with increasing effective temperature. Further, they span a wide range of Sc and Gd compositions, extending significantly beyond the limits observed in the pseudo‐binary ${\mathrm{Al}}_{1-x}{\mathrm{Gd}}_{x}N$ alloy.

Focusing on the 3500 K phase diagram as a representative case (Figure 1b), we observe that both metastable and stable wurtzite phases are accessible across a broad swath of composition space. Compositions with moderate Sc content and low to intermediate Gd content fall within these predicted regimes. These ranges are particularly promising for multifunctionality: ${\mathrm{Sc}}^{3+}$ and ${\mathrm{Gd}}^{3+}$ incorporation enable ferroelectricity in AlN, while ${\mathrm{Gd}}^{3+}$ may introduce magnetic behavior. The possibility of synthesizing such compositions as single‐phase wurtzite alloys expands the potential for realizing coupled ferroic behavior in a single‐material platform.

### 2.2 Realization of Quaternary ${\mathrm{Al}}_{1-x-y}{\mathrm{Sc}}_{x}{\mathrm{Gd}}_{y}N$ Thin Films

With such motivations in mind, we went on to synthesize ${\mathrm{Al}}_{1-x-y}{\mathrm{Sc}}_{x}{\mathrm{Gd}}_{y}N$ thin films with an intentional one‐dimensional (1D) composition gradient (as illustrated in Figure S2) using reactive co‐sputtering. We used p‐doped silicon (pSi) and platinized pSi substrates for structural and property investigation of the grown films. Leveraging our well‐parameterized synthesis routes for ${\mathrm{Al}}_{1-x}{\mathrm{Gd}}_{x}N$ [9, 14] and ${\mathrm{Al}}_{1-x}{\mathrm{Sc}}_{x}N$, [5, 22, 27] we tuned the growth conditions to achieve crystalline, quaternary ${\mathrm{Al}}_{1-x-y}{\mathrm{Sc}}_{x}{\mathrm{Gd}}_{y}N$ films by varying substrate temperatures (50–600 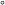), gas ratios ($N_{2}$:Ar = 1:9–9:1), and Gd magnetron power (25–150 W). Within this parameter space, we observed that higher growth temperatures (>400 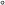) favored the wurtzite structure; thus, a substrate temperature of 500 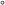, which is the highest temperature that reliably produces stable interfaces of the materials stack of films on platinized silicon, was chosen for all subsequent growths. The final series of films were grown under both $N_{2}$‐rich ($N_{2}$:Ar = 3:1) and Ar‐rich ($N_{2}$:Ar = 1:3) conditions on both single‐side polished pSi(100) and double‐side‐polished platinized pSi(100) substrates.

In terms of composition, we were driven by our optimistic theoretical predictions for wurtzite metastability to explore a relatively wide range of Sc‐ and Gd‐incorporation into AlN. Given the already complex parameter space inherent to sputter synthesis, our initial efforts for experimentally mapping out the AlN–ScN–GdN phase diagram employed ${\mathrm{Al}}_{0.7}{\mathrm{Sc}}_{0.3}$ or ${\mathrm{Al}}_{0.6}{\mathrm{Sc}}_{0.4}$ alloy targets to constrain the Sc content to either x≈0.2 or x≈0.3, respectively. The composition of several representative films was measured with electron probe microanalysis (EPMA) across the 1D compositional gradients to reveal that in both cases, the resulting quaternary ${\mathrm{Al}}_{1-x-y}{\mathrm{Sc}}_{x}{\mathrm{Gd}}_{y}N$ films do not necessarily have the target Al/Sc composition and that the Sc/(Al+Sc+Gd) ratio is invariable (x≈0.2 or x≈0.3 depending on the AlSc target composition) with Gd content; however, within the context of the current study we did not explore this further. In terms of Gd content, while initial synthesis of ${\mathrm{Al}}_{1-x-y}{\mathrm{Sc}}_{x}{\mathrm{Gd}}_{y}N$ prioritized modest incorporation, we ultimately explored Gd content up to y≈0.45 (as quantified by EPMA) by varying the Gd magnetron power. The final series of ${\mathrm{Al}}_{1-x-y}{\mathrm{Sc}}_{x}{\mathrm{Gd}}_{y}N$ films demonstrates a Sc content of either x≈0.2 or x≈0.3 and spans a wide range of Gd incorporation (0.01 ≲ *y* ≲ 0.45) in both cases. EPMA also probed the nitrogen content, expressed as the anion ratio N/(N+O), which was on average ≈0.87 for $N_{2}$‐rich growths and ≈0.85 for Ar‐rich growths (see Table S1). This is similar to what has been observed previously for ${\mathrm{Al}}_{1-x}{\mathrm{Gd}}_{x}N$ [14] and other nitride materials grown in the same deposition chamber [28].

**FIGURE 2:**
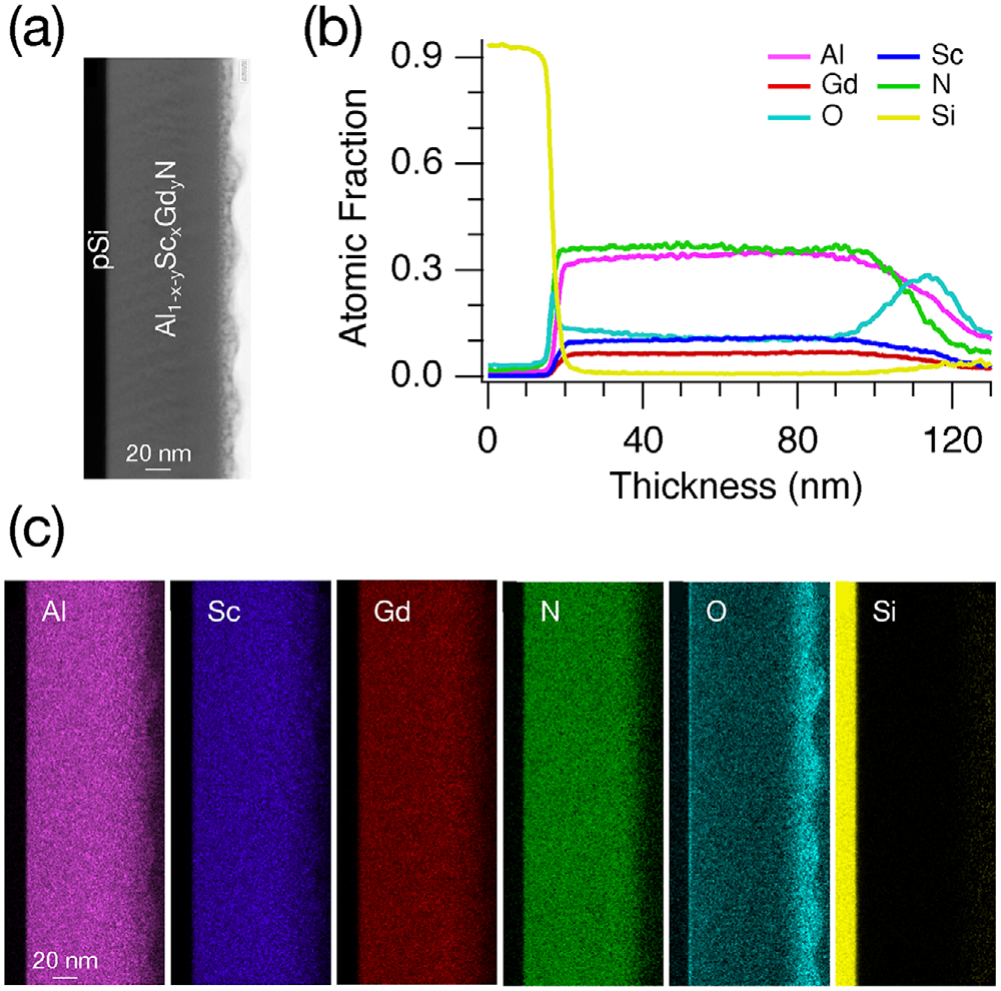
(a) STEM‐HAADF image; (b) EDS atomic % line profiles; and (c) EDS atomic % elemental maps of Al, Sc, N, O, and Si for a representative ${\mathrm{Al}}_{1-x-y}{\mathrm{Sc}}_{x}{\mathrm{Gd}}_{y}N$ film with Gd content *y* ≈0.25 and Sc content *y* ≈0.20. The EDS mapping demonstrates homogeneous distribution of Al, Sc, Gd, and N throughout the thickness of the film as well as some deleterious surface oxidation. The EDS line profiles further support that oxidation is primarily occurring at the surface.

Beyond composition, we investigated the microstructure and uniformity of a characteristic film with a Gd content of *y* ≈0.25 and Sc content *y* ≈0.20 (as measured by EMPA) via scanning transmission electron microscopy (STEM). The STEM‐HAADF image contained in (Figure 2)a reveals a film with somewhat columnar grains, which is typical for sputter‐deposited material. STEM‐EDS elemental mapping (Figure 2c) demonstrates homogeneous distribution of Al, Sc, Gd, and N throughout the thickness of the film, which is desired for piezoelectric and ferroelectric materials. It also reveals some surface oxidation of the films, as evidenced by the comparably brighter right‐side edge in the oxygen map of (c). This is further supported by the spike in the EDS line profile for oxygen at ∼115 nm from the left‐side edge of the cross‐section in (b) which corresponds to the surface of the film. This surface oxide layer contributes to the oxygen content measured by EPMA.

**FIGURE 3:**
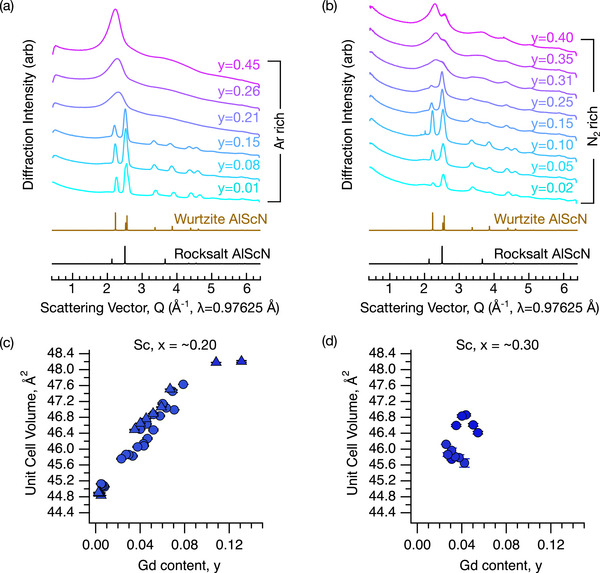
Integrated synchrotron GIWAXS data for ${\mathrm{Al}}_{1-x-y}{\mathrm{Sc}}_{x}{\mathrm{Gd}}_{y}N$ grown under (a) Ar‐rich and (b) $N_{2}$‐rich chamber conditions for Gd content *y* = 0.01 to 0.45. ${\mathrm{Al}}_{1-x-y}{\mathrm{Sc}}_{x}{\mathrm{Gd}}_{y}N$ crystallizes into a phase‐pure wurtzite structure at 0.01 ≲ *y* ≲ 0.15 which is well textured in the case of $N_{2}$‐rich growths or randomly oriented in the case of Ar‐rich growths. For $N_{2}$‐rich growths, coexistence of rocksalt and wurtzite phases occurs at *y* ≳ 0.15, and rocksalt dominates with increasing Gd content. Contrarily, Ar‐rich growth yields largely amorphous material for Gd content *y* ≳ 0.15. In addition, unit cell volumes for ${\mathrm{Al}}_{1-x-y}{\mathrm{Sc}}_{x}{\mathrm{Gd}}_{y}N$ extracted from LeBail fits of the integrated GIWAXS data increase linearly for Sc content (c) *x* ≈0.20 and (d) *x* ≈0.30, which further supports successful Gd incorporation.

We employed both lab‐based X‐ray diffraction (XRD) and synchrotron grazing incidence wide‐angle X‐ray scattering (GIWAXS) to probe crystallinity and structure as a function of composition and deposition parameters for the ${\mathrm{Al}}_{1-x-y}{\mathrm{Sc}}_{x}{\mathrm{Gd}}_{y}N$ films. Figure 3 presents integrated synchrotron GIWAXS data for films grown under both (a) Ar‐rich and (b) $N_{2}$‐rich conditions along with calculated patterns for wurtzite AlScN and rocksalt AlScN. The close *d*‐spacings of some of the wurtzite AlScN and rocksalt AlScN peaks as well as the gradual peak shifts due to lattice parameter changes with increasing Gd content *y* complicate successful phase identification; as such, analysis of both integrated GIWAXS patterns and corresponding 2D detector images in parallel was critical. As indicated in Figure 3b, for $N_{2}$‐rich growths, phase‐pure wurtzite material is achieved at Gd content 0.02≲ *y* ≲0.15; all peaks in the corresponding traces are relatively sharp and can be attributed to the wurtzite phase. This is supported by Figure S3b which contains a representative 2D detector image for a film from $N_{2}$‐rich growth with low Gd substitution (y = 0.02); it clearly lacks demonstrable rocksalt features. Figure S3b further reveals that such films appear highly textured while ${\mathrm{Al}}_{1-x-y}{\mathrm{Sc}}_{x}{\mathrm{Gd}}_{y}N$ films with Gd content ≳0.15, as in Figure S3a, demonstrate coexistence of textured wurtzite and randomly oriented rocksalt phases. The rocksalt phase dominates with increasing Gd content.

Beyond $N_{2}$‐rich growth, ${\mathrm{Al}}_{1-x-y}{\mathrm{Sc}}_{x}{\mathrm{Gd}}_{y}N$ films grown under Ar‐rich conditions with Gd content ≲0.15 also demonstrate phase‐pure wurtzite material but seem comparably less textured and more randomly oriented, as evidenced in Figure S3e. For Gd substitution above *y* ≈0.15 (Figure S3d), films grown under Ar‐rich conditions appear largely amorphous. This is supported by the peak broadening in Figure 3a for the traces corresponding to 0.21≲ *y* ≲0.45, which indicates significant disorder in the crystalline phase, consistent with loss of crystallinity (amorphous or nanocrystalline material).

Additionally, wurtzite unit cell volume and lattice parameters for the ${\mathrm{Al}}_{1-x-y}{\mathrm{Sc}}_{x}{\mathrm{Gd}}_{y}N$ films were extracted from Lebail fits of the integrated GIWAXS data in space group $P6_{3}mc$. Figure 3 also displays experimental unit cell volume as a function of Gd substitution for Sc contents c) *x* ≈0.20 and d) *x* ≈0.30. Recall that for phase exploration across a wide Gd substitution range, the Sc content was held roughly constant. Nonetheless, it is clear from Figure 3c that regardless of gas ratio during growth, for Sc content *y* ≈0.20 the unit cell volume increases somewhat linearly with Gd substitution as expected, indicating successful Gd incorporation. This is further evidenced by Figure S4 in which similar trends can be seen for lattice parameters a and c. Further, it appears that films with Sc content *x* ≈0.30, as in Figure 3d, may follow a similar trend, but exploration across a wider Gd substitution range would be critical to make robust correlations between unit cell volume and Gd content at these higher‐Sc compositions.

**FIGURE 4:**
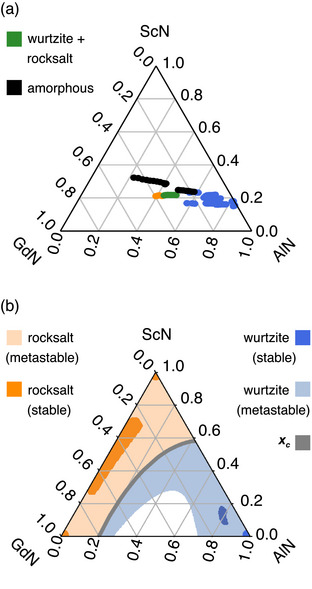
(a) Experimental ternary phase diagram for ${\mathrm{Al}}_{1-x-y}{\mathrm{Sc}}_{x}{\mathrm{Gd}}_{y}N$ films grown under both Ar‐ and $N_{2}$‐rich chamber conditions and (b) computationally‐predicted quaternary phase diagram of ${\mathrm{Al}}_{1-x-y}{\mathrm{Sc}}_{x}{\mathrm{Gd}}_{y}N$ at 3500 K, which is a reproduction of Figure 1b.

To summarize graphically, Figure 4a contains the first experimental exploration of phase boundaries for quaternary ${\mathrm{Al}}_{1-x-y}{\mathrm{Sc}}_{x}{\mathrm{Gd}}_{y}N$ grown from magnetron sputtering. As evidenced by the blue‐shaded data, it is apparent that sputter growth yield phase‐pure wurtzite material with up to *y* ≈0.15 Gd incorporation. For Gd content above *y* ≈0.15, it seems from Figure S5 that the resulting crystal phase for ${\mathrm{Al}}_{1-x-y}{\mathrm{Sc}}_{x}{\mathrm{Gd}}_{y}N$ films is strongly dependent upon $N_{2}$:Ar gas ratio. For films grown in a $N_{2}$‐rich atmosphere (Ar:$N_{2}$ = 1:3), we observe a coexistence of wurtzite and rocksalt phases within the 0.19 ≲ *y* ≲ 0.24 compositional range (green‐shaded data), above which the rocksalt phase dominates (orange‐shaded data). Contrarily, for films grown under Ar‐rich conditions (Ar:$N_{2}$ = 3:1), Gd substitution above *y* ≈0.15 yields largely amorphous material, as indicated by the black‐shaded data.

For comparison, Figure 4b contains our computationally‐predicted phase diagram for the alloyed ${\mathrm{Al}}_{1-x-y}{\mathrm{Sc}}_{x}{\mathrm{Gd}}_{y}N$ at an effective temperature of 3500 K, which is a reproduction of Figure 1h. The compositional range within which we experimentally observe stable or (*meta*)stable wurtzite‐structured material aligns with the computationally‐predicted regions of wurtzite stability. It also appears that the compositional range at which we experimentally see the wurtzite and rocksalt phases coexist aligns well with the predicted wurtzite/rocksalt phase boundary. Regardless of absolute ${\mathrm{Al}}_{1-x-y}{\mathrm{Sc}}_{x}{\mathrm{Gd}}_{y}N$ compositions, the trends in crystal phase as a function of Gd substitution at least loosely correlate with theoretical predictions. As such, our experimental studies may have occurred around an effective temperature of 3500 K; however, more robust studies are necessary to make stronger arguments in this regard. It should also be noted that the computational calculations were confined to crystalline ${\mathrm{Al}}_{1-x-y}{\mathrm{Sc}}_{x}{\mathrm{Gd}}_{y}N$, and phase explorations for amorphous materials are underway.

In concert, the data demonstrate that for ${\mathrm{Al}}_{1-y}{\mathrm{Sc}}_{0.23}{\mathrm{Gd}}_{y}N$ films sputter‐deposited within the parameter space reported herein, we can incorporate Gd up to *y* ≈0.15 and retain a phase‐pure wurtzite structure that is relatively well textured in the case of $N_{2}$‐rich chamber conditions. Further, films grown under $N_{2}$‐rich conditions with Gd substitution above *y* ≈0.15 demonstrate coexistence of textured wurtzite and polycrystalline rocksalt, and then the rocksalt phase dominates with increasing Gd content. On the contrary, we observe an onset of amorphization at *y* ≈0.21 for films grown under Ar‐rich conditions that is not observed in films from $N_{2}$‐rich growth.

### 2.3 Controlling Properties of AlN via Co‐Substitution With Sc and Gd

The successful demonstration of phase‐pure wurtzite ${\mathrm{Al}}_{1-x-y}{\mathrm{Sc}}_{x}{\mathrm{Gd}}_{y}N$ films with large incorporation of Sc and Gd is promising for the possibility of piezoelectric property enhancement. Here, we further investigate the effect of co‐substitution on bandgap, piezoelectricity, and potential for ferroelectricity.

#### 2.3.1 Optical Properties

**FIGURE 5:**
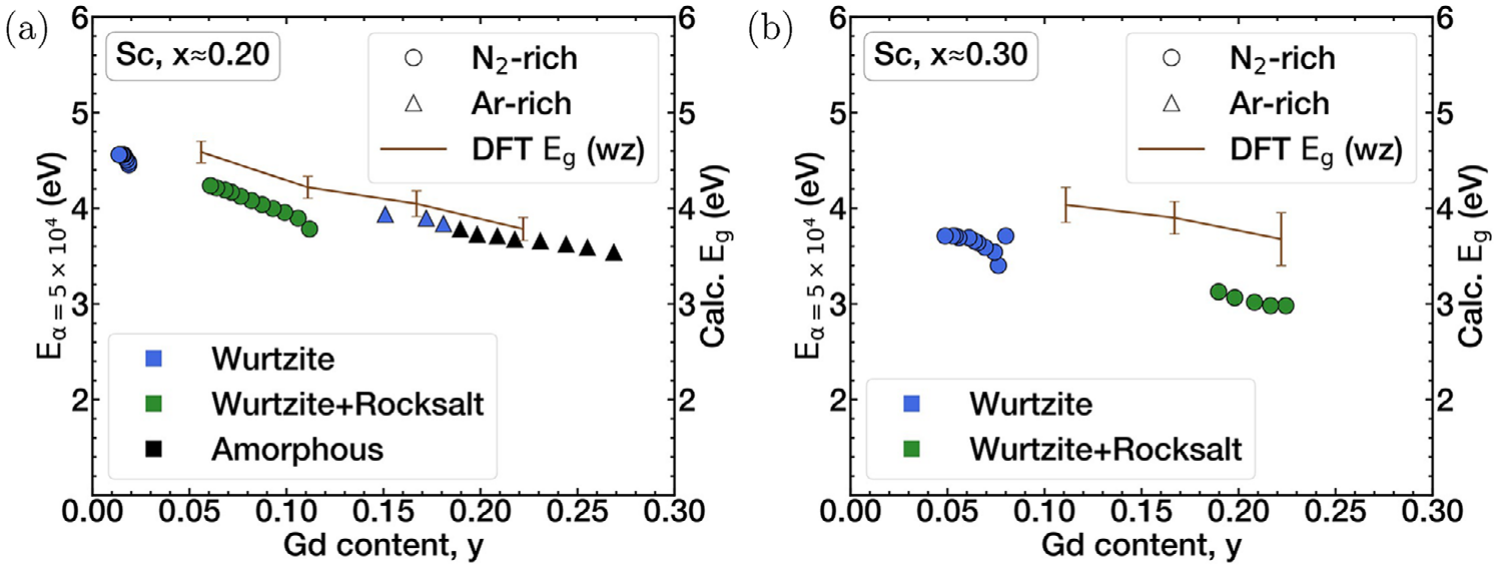
Ellipsometry combined with bandgap calculations show the material system is wide bandgap, and the gap is decreasing with increasing Gd concentration for (a) Sc≈0.20 and (b) Sc≈0.30. Both figures show trends of absorption onset energy ($E_{\alpha =5\times 10^{4}}$) for different synthesis parameters, compositions, and crystal structures of ${\mathrm{Al}}_{1-x-y}{\mathrm{Sc}}_{x}{\mathrm{Gd}}_{y}N$. The trends of $E_{\alpha =5\times 10^{4}}$ show comparable trends to the calculated bandgap of wurzite‐derived ${\mathrm{Al}}_{1-x-y}{\mathrm{Sc}}_{x}{\mathrm{Gd}}_{y}N$ ($E_{g}$ wurtzite) versus composition. These experimental and computational results demonstrate that ${\mathrm{Al}}_{1-x-y}{\mathrm{Sc}}_{x}{\mathrm{Gd}}_{y}N$ is a wide bandgap material system.

To investigate the optical properties, spectroscopic ellipsometry was performed on ${\mathrm{Al}}_{1-x-y}{\mathrm{Sc}}_{x}{\mathrm{Gd}}_{y}N$ films to extract the absorption coefficient (α, see Methods for details). The absorption coefficients for different compositions of ${\mathrm{Al}}_{1-x-y}{\mathrm{Sc}}_{x}{\mathrm{Gd}}_{y}N$ as a function of incident photon energy are shown in the Supporting Information (Figure S7). Due to the constraints of Tauc analysis for emerging, underexplored materials, we extracted the onset energy of the optical absorption ($E_{\alpha }$), which is closely related to the band gap, to identify trends across the composition space. For this, we chose an onset value of $\alpha =5\times 10^{4}$ ${\mathrm{cm}}^{-1}$; this value was also used in studies on similar materials [14, 15] and is discussed further in the Methods section. Figures 5a and 5b show the values of $E_{\alpha =5\times 10^{4}}$ extracted from Figure S7a,b, respectively. In parallel, band gaps were calculated for these alloys in the wurtzite structure and are shown in Figure 5. A clear trend of $E_{\alpha =5\times 10^{4}}$ decreasing with increasing Gd and Sc substitution is observed, matching the trend in the calculated bandgaps for varying Gd and Sc concentrations. This behavior was previously seen both experimentally and computationally in several members of the ${\mathrm{Al}}_{1-x}$ *RE* 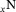 (*RE* = Gd, Tb, Pr) family, [14, 15] as well as in ${\mathrm{Al}}_{1-x}{\mathrm{Sc}}_{x}N$. [29] Despite the significant incorporation of Sc and Gd, these measurements support that this material system has a wide bandgap.

#### 2.3.2 Piezoelectric Properties

Single‐element substitution is an established method for designing ternary wurtzite alloys based on AlN [30, 31, 32, 33]. Furthermore, strong enhancement of piezoelectric properties is increasingly associated with the demonstration of ferroelectricity in AlN‐based material systems. [8, 30, 34] However, the effect of co‐substituting multiple elements into AlN upon piezoelectric properties remains elusive for the case of quaternary alloys like ${\mathrm{Al}}_{1-x-y}{\mathrm{Sc}}_{x}{\mathrm{Gd}}_{y}N$ — Will it be a simple linear combination of the two? Will there be any cooperative effects?

**FIGURE 6:**
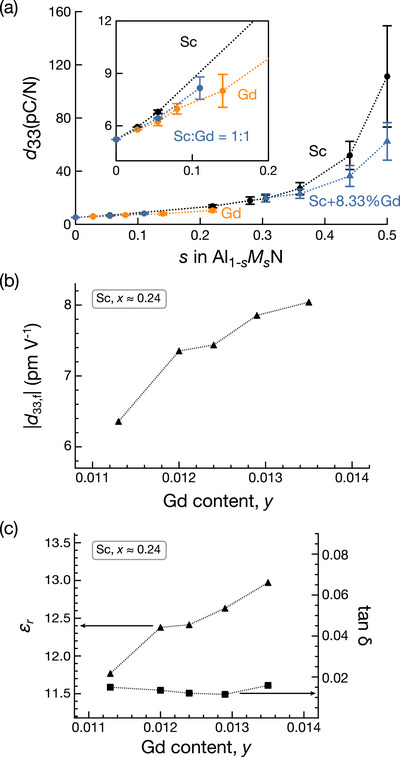
Longitudinal piezoelectric coefficient ($d_{33}$) as a function of total substitution fraction s. (a) Predicted $d_{33}$ of ${\mathrm{Al}}_{1-x}{\mathrm{Sc}}_{x}N$ (s = x), ${\mathrm{Al}}_{1-x}{\mathrm{Gd}}_{x}N$ (s = x), ${\mathrm{Al}}_{1-2x}{\mathrm{Sc}}_{x}{\mathrm{Gd}}_{x}N$ (s=2x) and ${\mathrm{Al}}_{1-x-0.083}{\mathrm{Sc}}_{x}{\mathrm{Gd}}_{0.083}N$ (s=x+0.083, blue triangles) alloys in the range s = 0.0 – 0.5. Inset highlights the low s region. The error bars are standard deviations across four random alloy configurations. (b) Experimental $d_{33}$ measured via DBLI for s=x+y≈0.25. (c) Permittivity (left axis, triangular markers) and loss tangent values (right axis, square markers) for s=x+y≈0.25.

To approach these questions, we performed a systematic computational study on both single‐element substitution (${\mathrm{Al}}_{1-x}{\mathrm{Sc}}_{x}N$ and ${\mathrm{Al}}_{1-x}{\mathrm{Gd}}_{x}N$) and co‐substitution (${\mathrm{Al}}_{1-x-y}{\mathrm{Sc}}_{x}{\mathrm{Gd}}_{y}N$). We calculated $d_{33}$ using linear response theory and DFT (see Methods for details). Figure 6a shows the predicted $d_{33}$; the results of single‐element substitution are the references for the discussion on co‐substitution. The results for ${\mathrm{Al}}_{1-x}{\mathrm{Sc}}_{x}N$ are consistent with previous work and serve as a benchmark [35]. $d_{33}$ increases linearly at low Sc composition (x≲ 0.2) but increases faster with larger Sc composition, especially as it approaches its critical composition around $x_{c}\approx 0.56$. The $d_{33}$ enhancement peaks out at the critical composition where the rocksalt phase starts appearing. ${\mathrm{Al}}_{1-x}{\mathrm{Gd}}_{x}N$ alloys (x≳ 0.1) were successfully demonstrated to be ferroelectrics [9], but their piezoelectric properties were not investigated. Figure 6a shows that Gd substitution increases $d_{33}$ linearly within the studied range (x < 0.22) and the results support the correlation between enhancement in piezoelectricity and realization of ferroelectricity discussed earlier. Lastly, comparing Gd to Sc substitution, we find that Sc substitution is more effective in increasing $d_{33}$ and the difference gets larger at higher alloying content. This is especially true when the Sc content approaches its critical composition $x_{c}$. The behavior can be explained by the distance in composition space to the critical composition since $d_{33}$ is well‐known to increase drastically near the morphotropic phase boundary [36]. Since the Sc critical composition $x_{c}\approx$ 0.56 is much lower than the Gd critical composition $y_{c}\approx$ 0.80 (see Figure 1), a larger enhancement for Sc substitution is expected for the discussed composition range.

Next, we computationally investigated the effect of co‐substitution on $d_{33}$ of wurtzite AlN‐based alloys. We started with equal amounts of Sc and Gd at a low total substitution fraction (s< 0.2; see inset of Figure 6a). We found that $d_{33}$ of ${\mathrm{Al}}_{1-x-y}{\mathrm{Sc}}_{x}{\mathrm{Gd}}_{y}N$ generally falls between those of ${\mathrm{Al}}_{1-x}{\mathrm{Sc}}_{x}N$ and ${\mathrm{Al}}_{1-x}{\mathrm{Gd}}_{x}N$. This suggests no cooperative effect for ${\mathrm{Al}}_{1-x-y}{\mathrm{Sc}}_{x}{\mathrm{Gd}}_{y}N$ at the low composition range. Figure 6b shows the scenario at higher total substitution fractions. We focus on the case when Gd composition y is fixed at around 0.083 and compare it to ${\mathrm{Al}}_{1-x}{\mathrm{Sc}}_{x}N$ with the same respective total substitution. We find that adding Gd to ${\mathrm{Al}}_{1-x}{\mathrm{Sc}}_{x}N$ increases $d_{33}$ but that the enhancement is considerably less than adding the same amount of Sc. This suggests that Gd substitution is less effective in increasing $d_{33}$ and no cooperative effect exists. However, in the situation when adding more Gd is experimentally easier than adding more Sc, turning to quaternary alloys like ${\mathrm{Al}}_{1-x-y}{\mathrm{Sc}}_{x}{\mathrm{Gd}}_{y}N$ can be an approach to further increase $d_{33}$.

To corroborate the predictions, investigate the piezoelectric nature of the films, and further confirm that a non‐centrosymmetric wurtzite‐derived crystal structure describes ${\mathrm{Al}}_{1-x-y}{\mathrm{Sc}}_{x}{\mathrm{Gd}}_{y}N$ alloys, small signal piezoelectric coefficient measurements were performed using a double beam laser interferometer (DBLI, see Methods for details). Figure 6b shows the magnitude of the effective piezoelectric coefficients ($|d_{33,f}|$) as a function of Gd content y for ${\mathrm{Al}}_{1-x-y}{\mathrm{Sc}}_{x}{\mathrm{Gd}}_{y}N$ films with Sc content of x≈0.24. Clear non‐zero piezoelectric coefficient values emerged from the measurements, further confirming the material can be described by a non‐centrosymmetric crystal structure. The values of $|d_{33,f}|$ in Figure 6b show an increase with increasing Gd concentration, similar to other substituted AlN‐based thin films [33, 37, 38, 39, 40, 41], which indicates that the co‐substitution of Gd and Sc is a promising strategy for improving piezoelectric response. This approach is more effective in a Sc rich composition where the rocksalt phase starts to appear because co‐alloying enables more total substitution as discussed in Figure1. The scientific origin of the improvement in $|d_{33,f}|$ has been attributed to the “frustration” of this system – ${\mathrm{Al}}^{3+}$ prefers fourfold coordination and ${\mathrm{Sc}}^{3+}$ and ${\mathrm{Gd}}^{3+}$ prefer sixfold coordination [30, 42, 43, 44]. In addition, in our previous report, we showed that the large ionic radius difference between ${\mathrm{Al}}^{3+}$ and ${\mathrm{Gd}}^{3+}$ causes local distortion and switching barrier reduction [9], which may contribute to the ionic displacement under an electric field and resultant dielectric and piezoelectric response. Values of $|d_{33,f}|$ depend on material quality, as seen in the well‐studied ${\mathrm{Al}}_{1-x}{\mathrm{Sc}}_{x}N$ system [45, 46, 47]. Importantly, Figure 6b shows that a small addition of Gd leads to a significant increase in $|d_{33,f}|$, although the values of $|d_{33,f}|$ are comparable to reported ${\mathrm{Al}}_{1-x}{\mathrm{Sc}}_{x}N$ with x≈0.24 (6 – 12 pm V^−1^) due to the lack of process optimization in this study. This result suggests that both Gd and Sc can improve the piezoelectric property simultaneously, supporting the claims of predicted increases in $d_{33}$ in Figure 6a,b. Moreover, Sc/Gd co‐alloying, which can incorporate more substitution in total as demonstrated in this study for the first time, is a promising strategy to breakthrough the relatively small piezoelectric response of wurtzite materials.

Figure 6c shows that the values of relative permittivity ($\varepsilon_{r}$), extracted using capacitance measurements, increase with increasing Gd concentration, y. This is attributed to a lattice softening leading to an increase in the ionic contribution to $\varepsilon_{r}$ [42, 48, 49]. A greater ionic contribution to $\varepsilon_{r}$ supports the improvement of the piezoelectric responses discussed above. Figure 6c also shows values of tan(δ)<0.02 for all values of Gd concentration, indicating insulating behavior. The trends of $\varepsilon_{r}$, tan(δ), and $d_{33,f}$ all show piezoelectric insulating behavior typical of substituted AlN‐based wurtzite materials and consistent with the measured and predicted bandgap. Measurement of ${\mathrm{Al}}_{1-x-y}{\mathrm{Sc}}_{x}{\mathrm{Gd}}_{y}N$ with higher Gd was complicated by polycrystalline microstructure and rocksalt phase impurities (see Figure S6).

### 2.4 Predicted and Realized Polarization Switching in ${\mathrm{Al}}_{1-x-y}{\mathrm{Sc}}_{x}{\mathrm{Gd}}_{y}N$

The natural next step of the strategy we employ to design functional properties in AlN‐based alloys is to examine whether ${\mathrm{Al}}_{1-x-y}{\mathrm{Sc}}_{x}{\mathrm{Gd}}_{y}N$ alloys are ferroelectric. Since device optimization remains challenging for quaternary alloys, experimental demonstration will be a future focus. Here, we provide strong computational evidence in addition to the enhancement of piezoelectricity discussed above — consistent with a change in the predicted switching mechanisms at atomic scale. While enhancement in piezoelectricity is generally associated with the demonstration of ferroelectricity for wurtzite AlN‐based alloys, strong evidence is needed to support that ${\mathrm{Al}}_{1-x-y}{\mathrm{Sc}}_{x}{\mathrm{Gd}}_{y}N$ alloys can exhibit ferroelectric properties. Building on our previous finding that connects DFT calculations on the atomic scale to experimental observations of polarization switching at room temperature for wurtzite ferroelectrics [9, 50], we further investigate the minimal total substitution fraction needed to cause a change in the switching mechanism.

**FIGURE 7:**
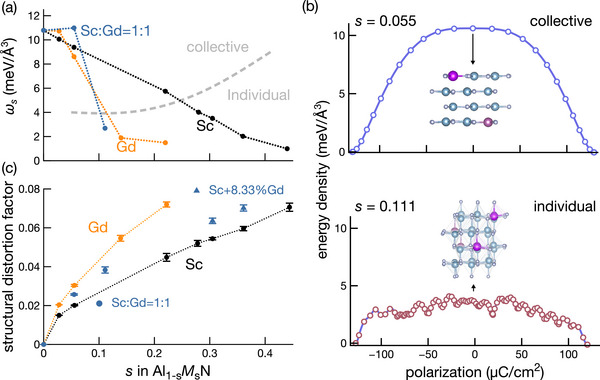
Polarization switching in ${\mathrm{Al}}_{1-x-y}{\mathrm{Sc}}_{x}{\mathrm{Gd}}_{y}N$ alloys. (a) Switching barrier $\omega_{s}$ as a function of total substitution fraction s. Results for ${\mathrm{Al}}_{1-x}{\mathrm{Sc}}_{x}N$ and ${\mathrm{Al}}_{1-x}{\mathrm{Gd}}_{x}N$ are taken from Refs. [9, 50]. The gray dashed line represents the approximate separation of the switching mechanisms. (b) Polarization switching pathway and nonpolar structures for s = 0.055 and 0.111. (c) Structure distortion factor as a function of total substitution fraction s in ${\mathrm{Al}}_{1-x}{\mathrm{Sc}}_{x}N$ (s=x), ${\mathrm{Al}}_{1-x}{\mathrm{Gd}}_{x}N$ (s=x), and ${\mathrm{Al}}_{1-x-y}{\mathrm{Sc}}_{x}{\mathrm{Gd}}_{y}N$ alloys (s=x+y). Blue circles represent alloys where x=y in ${\mathrm{Al}}_{1-x-y}{\mathrm{Sc}}_{x}{\mathrm{Gd}}_{y}N$ alloys, and blue triangles correspond to ${\mathrm{Al}}_{1-x-y}{\mathrm{Sc}}_{x}{\mathrm{Gd}}_{y}N$ with fixed Gd composition (y=0.0833). Error bars are standard deviations across four alloy configurations.

Our previous works showed that the predicted minimal substitution fractions to induce ferroelectric switching for Sc and Gd ternary alloys are ≲0.28 and ≲0.14, respectively, and these values are consistent with the typical minimal substitution fraction for ferroelectricity to be observed in ${\mathrm{Al}}_{1-x}{\mathrm{Sc}}_{x}N$ and ${\mathrm{Al}}_{1-x}{\mathrm{Gd}}_{x}N$ experimentally. [9, 50] Figure 7a shows the calculated switching barrier as a function of total substitution fraction s for ${\mathrm{Al}}_{1-x-y}{\mathrm{Sc}}_{x}{\mathrm{Gd}}_{y}N$ alloys with an equal amount of Sc and Gd, along with the calculated switching barriers for ${\mathrm{Al}}_{1-x}{\mathrm{Sc}}_{x}N$ and ${\mathrm{Al}}_{1-x}{\mathrm{Gd}}_{x}N$ for comparison. The barrier drops drastically from s= 0.055 to s= 0.111; this drop is associated with the change in switching mechanism (see Figure 7b). The polarization switching pathway is smooth for s= 0.055 and follows the known collective switching mechanism with a layered hexagonal structure that is nonpolar. In comparison, s= 0.111 has a very rugged switching pathway, and the associated nonpolar structure has half of the cation‐centered tetrahedra pointing up and the other half pointing down. The finding suggests that a significantly smaller total substitution fraction is needed to make ferroelectric ${\mathrm{Al}}_{1-x-y}{\mathrm{Sc}}_{x}{\mathrm{Gd}}_{y}N$ alloys than the value for ${\mathrm{Al}}_{1-x}{\mathrm{Sc}}_{x}N$, which is noteworthy given the impressive properties of ${\mathrm{Al}}_{1-x}{\mathrm{Sc}}_{x}N$; this may become a viable strategy to improve its commercializability given the high cost of Sc. The required s is comparable to that of ${\mathrm{Al}}_{1-x}{\mathrm{Gd}}_{x}N$ given that they are not distinguishable for the composition resolution.

We previously showed that the s required to induce a change in switching mechanisms at the atomic scale in wurtzite materials is qualitatively correlated with the extent of structural frustration caused by substitution [9, 50]. Distributions of Al‐N bond lengths and Al‐N‐Al angles are commonly used to compare both substitution type and amount. Here, we defined a single value, the structural distortion factor (SDF; see Methods for details), for each composition to facilitate qualitative comparison. Figure 7c shows the structural distortion factor for ${\mathrm{Al}}_{1-x}{\mathrm{Sc}}_{x}N$, ${\mathrm{Al}}_{1-x}{\mathrm{Gd}}_{x}N$, and ${\mathrm{Al}}_{1-x-y}{\mathrm{Sc}}_{x}{\mathrm{Gd}}_{y}N$ alloys. Consistent with the predicted results for piezoelectric behavior as a function of composition (see Figure 6), the SDFs of ${\mathrm{Al}}_{1-x-y}{\mathrm{Sc}}_{x}{\mathrm{Gd}}_{y}N$ alloys fall between those of ${\mathrm{Al}}_{1-x}{\mathrm{Sc}}_{x}N$ and ${\mathrm{Al}}_{1-x}{\mathrm{Gd}}_{x}N$ alloys. Incorporating Gd into ${\mathrm{Al}}_{1-x}{\mathrm{Sc}}_{x}N$ alloys drastically increases the SDF for the same total substitution fraction. Starting with ${\mathrm{Al}}_{0.972}{\mathrm{Sc}}_{0.028}N$, adding another fraction of x=0.028 Sc only increases the SDF by about 34% while adding a fraction of y=0.028 Gd increases the SDF by about 73%. This offers some rationale for our prediction that less total substitution fraction is needed to induce the change in switching mechanism for ${\mathrm{Al}}_{1-x-y}{\mathrm{Sc}}_{x}{\mathrm{Gd}}_{y}N$ alloys in comparison to ${\mathrm{Al}}_{1-x}{\mathrm{Sc}}_{x}N$ alloys.

**FIGURE 8:**
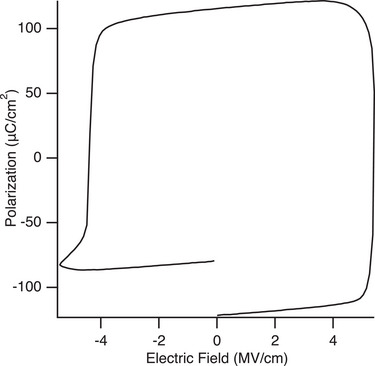
Polarization ‐ electric field hysteresis loop in ${\mathrm{Al}}_{0.83}{\mathrm{Sc}}_{0.15}{\mathrm{Gd}}_{0.02}N$ film shows unambiguous ferroelectric hysteresis.

To demonstrate the ferroelectric switching in the deposited ${\mathrm{Al}}_{1-x-y}{\mathrm{Sc}}_{x}{\mathrm{Gd}}_{y}N$ films, we conducted polarization ‐ electric field loop measurements (see Methods). Unambiguous ferroelectric switching and resultant hysteresis are observed in ${\mathrm{Al}}_{0.83}{\mathrm{Sc}}_{0.15}{\mathrm{Gd}}_{0.02}N$ film as seen in Figure 8. The average coercive field of the co‐alloyed film (($E_{c}$+(‐$E_{c}$))/2 = 4.9 MV cm^−1^) is comparable to ${\mathrm{Al}}_{0.7}{\mathrm{Sc}}_{0.3}N$ films deposited using the same chamber (4.4–5.2 MV cm^−1^) [5] even with the less Sc content in the co‐alloyed film, which implies that Gd contributes to coercive field reduction. This result supports the DFT simulations discussed above.

### 2.5 Outlook

We posit that quaternary nitride alloys based on wurtzite AlN represent a promising platform to realize single‐phase multiferroics that can smoothly integrate with conventional microelectronics. However, significant fundamental investigation will be required to realize this goal. One key challenge of this route toward single‐phase multiferroics lies in synthesizing quaternary nitride thin‐film materials with high enough alloying content to engender such desired properties. Our thermodynamic calculations and synthesis of quaternary ${\mathrm{Al}}_{1-x-y}{\mathrm{Sc}}_{x}{\mathrm{Gd}}_{y}N$ with significant amounts of both Sc and Gd, as well as our experimental demonstration of piezoelectricity in these films, therefore represent a significant step toward initial fundamental understanding of this new phase space. Excitingly, we predict and experimentally demonstrate in this work that ${\mathrm{Al}}_{1-x-y}{\mathrm{Sc}}_{x}{\mathrm{Gd}}_{y}N$ should be able to display piezoelectricity and ferroelectric switching with sufficient optimization of synthesis and properties.

Beyond ferroelectricity, inducing long‐range magnetic order is critical for a magneto‐electric multiferroic. The Al sublattice forms a hexagonal close‐packed structure, which has a percolation threshold of 0.195. This implies that a substitution of at least ∼0.2 of a magnetic cation may lead to magnetic order. The highest Gd substitution that we have achieved here is y≈ 0.15, just under the relevant percolation threshold. Looking at the thermodynamics of these heterostructural alloy systems, we speculate that even higher effective temperatures will be required to stabilize higher levels of Gd incorporation into the wurtzite phase, which may be necessary to induce magnetic ordering. We recently measured the magnetic properties of a ferroelectric film of ${\mathrm{Al}}_{1-y}{\mathrm{Gd}}_{y}N$ with y=0.14, and it displayed paramagnetic behavior at temperatures as low as T=2 K but did not exhibit behavior consistent with long‐range magnetic ordering [9]. As such, we speculate that achieving higher Gd substitution into the wurtzite structure will be necessary to realize the simultaneous coexistence of ferroelectric properties and magnetic order that is characteristic of a multiferroic material.

## 3 Conclusion

Presently, there persists a critical need for more energy‐efficient devices for a range of applications including computing and data storage, sensing, and resonators. This must be addressed by the fundamental discovery and characterization of new, stable functional materials, and quaternary alloys are an emerging frontier of research. We tackle this challenge using a combined computational and experimental study on a novel quaternary alloy based on wurtzite AlN: ${\mathrm{Al}}_{1-x-y}{\mathrm{Sc}}_{x}{\mathrm{Gd}}_{y}N$, employing computational thermodynamics and combinatorial thin‐film growth via reactive magnetron sputtering. The predicted phase diagrams suggest that a higher total substitution fraction (s=x+y) is plausible at an effective temperature comparable to that for ternary ${\mathrm{Al}}_{1-x}{\mathrm{Sc}}_{x}N$ and ${\mathrm{Al}}_{1-x}{\mathrm{Gd}}_{x}N$ alloys, which we successfully realized previously. We support these predictions experimentally by identifying and parameterizing recipes for sputter growth of wurtzite ${\mathrm{Al}}_{1-x-y}{\mathrm{Sc}}_{x}{\mathrm{Gd}}_{y}N$ thin films with a high substitution fraction s=x+y< 0.35, as verified by synchrotron grazing incidence wide‐angle X‐ray scattering.

After successful demonstration of the synthesis of ${\mathrm{Al}}_{1-x-y}{\mathrm{Sc}}_{x}{\mathrm{Gd}}_{y}N$ presented herein, we characterized the bandgap and piezoelectric coefficient $|d_{33,f}|$ of these films and found good consistency between the experimental results and computational predictions. ${\mathrm{Al}}_{1-x-y}{\mathrm{Sc}}_{x}{\mathrm{Gd}}_{y}N$ alloys hold promise as functional piezoelectric materials and future ferroelectric materials. Gd substitution is more effective than Sc substitution at inducing a change in the polarization switching mechanism, consistent with our prior results on the ternary alloys [9]. This offers a potential path toward cheaper devices because Gd is significantly less expensive than Sc. Here, we demonstrated preliminary ferroelectric switching of an ${\mathrm{Al}}_{0.83}{\mathrm{Sc}}_{0.15}{\mathrm{Gd}}_{0.02}N$ film; however, device optimization remains to be performed. Nonetheless, we provide strong computational evidence that ${\mathrm{Al}}_{1-x-y}{\mathrm{Sc}}_{x}{\mathrm{Gd}}_{y}N$ can be ferroelectric at even low total substitution fraction. This is based on the predicted polarization switching mechanisms at atomic scale and significant enhancement in piezoelectricity. Overall, our work sets the foundation toward wurtzite‐nitride‐based multiferroics.

## 4 Methods

### 4.1 Experimental Methods

Experimental data used by this study had been analyzed using the COMBIgor software package [51] and are publicly available in the National Renewable Energy Laboratory (NREL) high‐throughput experimental materials database at https://htem.nrel.gov [52].

#### 4.1.1 Synthesis

Combinatorial thin films of ${\mathrm{Al}}_{1-x-y}{\mathrm{Sc}}_{x}{\mathrm{Gd}}_{y}N$ were deposited using RF cosputtering from elemental and alloy targets on 3'’ magnetrons in a vacuum sputter chamber with a base pressure <5 × 10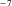 Torr. The magnetrons were in a standard balanced magnetron configuration, had a working distance of 165 mm, and were at a 120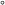  angle to each other. The $pO_{2}$ was <2 × 10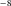 Torr and the $pH_{2}O$ was <3 × 10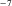 Torr at 500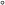 C. The powers used were 300 W (${\mathrm{Al}}_{0.7}{\mathrm{Sc}}_{0.3}$, 99.9% or ${\mathrm{Al}}_{0.6}{\mathrm{Sc}}_{0.4}$, 99.9%) and 25–150 W (Gd, 99.5%) in a stational substrate geometry, yielding a compositional gradient of 0.01 > *y* > 0.47. Deposition occurred at a total chamber pressure of 4 mTorr and the substrate was heated to Tsub = 500 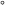. Some depositions occurred under 5 sccm of Ar and 15 sccm of $N_{2}$ (99.999%) gases while others occured under 15 sccm of Ar and 5 sccm of $N_{2}$ gases (introduced near the targets). For depositions under 5:15 Ar:N, the targets were in a poisoned regime during the growth. The targets were presputtered for 60 min with the substrate shutter closed, followed by a 60 min deposition with an approximate deposition rate of 3 nm min^−1^. Films were grown on 50.8 × 50.8 mm single‐side polished pSi(100) and double‐side polished p‐PtSi substrates with a native oxide layer (3 nm); no substrate pretreatment was performed.

#### 4.1.2 Composition and Structural Characterization

Cation composition was measured with X‐ray fluorescence (XRF) using a Bruker M4 Tornado under vacuum (∼15 Torr). The average elemental composition of select samples was analyzed at the University of Oregon Center for Advanced Materials Characterization in Oregon (CAMCOR) using a Cameca SX100 electron probe microanalyzer (EPMA) with wavelength dispersive spectrometry (WDS). All sites on samples and standards were measured with a 5 μm wide beam with 30 nA of current at incident electron beam energies of 10, 15, and 20 keV. Raw k‐ratios were then imported into STRATAGem thin film processing software to quantify thin film compositions. The XRF data was calibrated using the EPMA data. For samples grown on platinized pSi substrates, the composition was assumed to be the same as pSi witness substrates.

Scanning transmission electron microscopy (STEM) high‐angle annular dark‐field (HAADF) images were acquired with a Thermo Fisher Scientific Spectra 200 transmission electron microscope operating at an accelerating voltage of 200 keV, probe current of 200–250 pA, convergence angle of 24.2 mrad, and approximate probe size of 0.1 nm. Specimens for TEM were prepared from a selected region via in situ focused ion beam (FIB) lift‐out methods using a Tescan Solaris Ga FIB‐SEM workstation. [53] Elemental mapping was performed in STEM mode using the Super‐X EDS system equipped with four windowless silicon drift detectors, allowing for high count rates and chemical sensitivity (down to 0.5–1 at.%). The EDS data were quantified using a multi‐polynomial parabolic background and absorption correction in Velox.

Laboratory X‐ray diffraction (XRD) patterns were collected with Cu $K_{\alpha }$ radiation on a Bruker D8 Discover diffractometer that allowed spatial mapping across the compositionally‐graded thin films. Synchrotron grazing incidence wide angle X‐ray scattering measurements were performed on select samples at beamline 11‐3 at the Stanford Synchrotron Radiation Lightsource, SLAC National Accelerator Laboratory. The data were collected with a Rayonix 225 area detector at room temperature using a wavelength of λ = 0.97625 Å, 1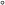  and 3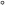  incident angles, a 150 mm sample‐to‐detector distance, and a beam size of approximately 50 μm vertical × 150 μm horizonal. The detector images were calibrated with a ${\mathrm{LaB}}_{6}$ standard, integrated with the Nika and Irena SAS packages using Igor Pro [54, 55], and processed with PyFAI and pygix [56, 57]. Integrated data were averaged from five frames of 15 s each. Obvious outlier peaks from hot pixels were smoothed out manually. LeBail fits of integrated plots were performed using the GSAS‐II software suite [58] in space group $P6_{3}mc$ by refining lattice parameters and sample displacements.

#### 4.1.3 Optical Characterization

Spectroscopic ellipsometry data were acquired at 65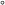 and 75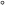 incident angles on single rows of select combinatorial samples (11 points per row) using a J.A. Woollam Co. M‐2000 variable angle ellipsometer. The samples were grown on crystalline pSi(100) substrates, with a native silicon oxide layer. The range of film thicknesses was approximately 70 – 300 nm, allowing for effective capture of the optical properties of the film.

Complete EASE software was used to model the data by fitting the real and imaginary parts of the dielectric function with a four‐layer model consisting of the silicon substrate, native silicon oxide, the ${\mathrm{Al}}_{1-x-y}{\mathrm{Sc}}_{x}{\mathrm{Gd}}_{y}N$ films, and surface roughness approximated with a standard mixed film/void Bruggeman Effective Medium Approximation (EMA) layer. The silicon and native silicon oxide were modeled using well‐known optical constants provided by the Complete EASE software. All the ${\mathrm{Al}}_{1-x-y}{\mathrm{Sc}}_{x}{\mathrm{Gd}}_{y}N$ film layers were modeled using polynominal spline‐based, Kramers–Kronig‐consistent, parametric semiconductor oscillator model (Psemi‐M0) — which are established models for accurately fitting materials without a well‐known bandgap where maximum flexibility is needed. The values of α in Figure S7 were extracted from this four‐layer model.

The absorption‐edge energy was determined at $\alpha =5\times 10^{4}$ ${\mathrm{cm}}^{-1}$ for multiple reasons. The main reason was pragmatic: previous reports on nitride thin films used the same value, which promotes consistent comparison between studies [14, 15]. The second reason for choosing this value pertains to sufficient penetration into the film being required to capture the properties of the bulk of the material. This α value corresponds to a penetration depth of 200 nm. Given that the range of film thicknesses was approximately 70 – 300 nm, a penetration depth of 200 nm sufficiently captures the bulk optical properties of the measured films, and gives a metric useful for characterization in applications such as solar cells [59]. A third reason was that this absorption onset is sufficiently above mid‐gap states that may generate Urbach tails such as the tail seen in Figure S7b.

#### 4.1.4 DBLI Methods

Small signal piezoelectric coefficient measurements were collected using an aixacct aixDBLI research line double beam laser interferometer (DBLI) instrument. First, a quartz reference was put into the instrument to calibrate the measured $d_{33}$ against the known $d_{33}$ of the quartz reference. Next, the incident top and bottom laser beams were aligned to be concentric. The reference beam and measurement beam were then adjusted such that the center of an interference pattern between the two beams was centered on the optical intensity detector of the instrument. The intensities of the reference and measurement beams were set equal after alignment with the detector. Minor adjustments were made to the intensities and positions of the reference and measurement beams until the measured $d_{33}$ matched the known $d_{33}$ of the quartz. The quartz reference was then removed, and the film sample was put into the instrument. Minor adjustments were made to the position and intensity of the reference and measurement beams to account for the difference in tilt and reflectivity of the samples relative to the quartz reference.

The measured ${\mathrm{Al}}_{1-x-y}{\mathrm{Sc}}_{x}{\mathrm{Gd}}_{y}N$ thin films were sputtered onto double side polished Si. The back side of the Si was coated with platinum to create a surface as reflective as possible. This Pt coating was used to eliminate calibration errors arising from Si not reflecting well at the 628 nm wavelength of the He‐Ne laser in the DBLI. The DBLI measurements were made on as‐grown electrodes and film, without any heating cycles or large signal electric fields applied to the sample that might lead to changes in the material properties.

To calculate the piezoelectric coefficient of the film unstrained by the Si substrate ($d_{33,f}$) from the as‐measured piezoelectric coefficient ($d_{33,f,meas}$), we used the method pioneered by Sivaramakrishnan et al. to convert $d_{33,f,meas}$ to $d_{33,f}$ [60, 61]. This involved measuring $d_{33,f,meas}$ on two different electrode sizes to account for changes in mechanical properties as a function of composition and using Equation (15) in Ref. [61] to calculate $d_{33,f}$. The two electrodes used were 200 and 300 μm diameter circles. Note that $d_{33,f}$ is an effective piezoelectric coefficient that includes substrate clamping effects, which is not the intrinsic piezoelectric coefficient ($d_{33}$).

To displace the film and observe the piezoelectric effect, a small signal sinusoidal E‐field was applied to the films with a frequency of 200 Hz and a peak amplitude of 15 V ($V_{ss}$). The displacement measured during a single 200 Hz cycle was averaged for 1000 cycles (which equates to a 5 s measurement). A total of ten 1000‐cycle measurements were taken then averaged to obtain a final value of $d_{33,f,meas}$. This averaging was done to improve the signal‐to‐noise ratio of the measurement. The lock‐in amplifier that is part of the aixDBLI was also used to improve the signal‐to‐noise ratio.

Loss tangent and electrical permittivity measurements were made simultaneous to the DBLI small signal measurement and used the same excitation signal. Therefore, the same small signal electrical settings apply to the tan(δ) and $\varepsilon_{r}$ measurements — 15 V, 200 Hz, 10 points + 1000 cycles of the 200 Hz signal.

#### 4.1.5 Ferroelectric Characterization

Polarization ‐ electric field loops were measured using a Radiant ferroelectric tester on Au/Ti/(Al,Sc,Gd)N/Pt/Si capacitors. The size of the Au/Ti top electrodes is 50 um in diameter. A triangular electric field with 10 kHz was applied from the bottom Pt electrode, and charge flow was collected from the Au/Ti top electrode.

### 4.2 Computational Methods

#### 4.2.1 DFT Parameters

We performed density functional theory (DFT) calculations to predict thermodynamics of ${\mathrm{Al}}_{1-x-y}{\mathrm{Sc}}_{x}{\mathrm{Gd}}_{y}N$ alloys and their properties in response to an electric field. All the calculations were executed using the Vienna Ab‐initio Simulation Package (VASP 5.4.4) [62, 63]. The Perdew‐Burke‐Ernzerhof (PBE) exchange‐correlation functional [64] was used and an on‐site Hubbard potential U = 3.0 eV was applied to the Sc d orbitals. The core electrons were described by the projector augmented wave (PAW) pseudopotentials [65] and the specific pseudopotentials used in this work are: Al 04Jan2001, Sc_sv 07Sep2000, Gd_3 06Sep2000, and N_s 07Sep2000. The wavefunctions were expanded as plane waves with a kinetic energy cutoff of 340 eV and the Brillouin zone was sampled using automatically generated Γ‐centered Monkhorst‐Pack k‐point grids with length ($R_{k}$) of 20. Self‐consistency was ensured by convergence criteria of $10^{-6}$ eV. The same set of parameters were used unless specified.

#### 4.2.2 AlN–ScN–GdN Ternary Phase Diagram

The AlN–ScN–GdN phase diagram was calculated from the Gibbs free energy of mixing ($\Delta G_{mix}$),

(1) $$\Delta G_{mix}=\Delta H_{mix}-T\Delta S_{mix}$$

where, the mixing enthalpy ($\Delta H_{mix}$) is calculated from DFT total energy and the regular solution model. [66, 67] Ideal mixing was used for configurational entropy. Vibrational entropy was not included assuming cancellation between reactants (binary compounds) and products (alloys). For mixing enthalpy, we used the special quasi‐random structure (SQS) approach [68], implemented in ATAT [69], to model alloys with a random distribution of Al, Gd, and Sc at the high temperature limit. A 96‐atom supercell was used. Based on fully relaxed structures of AlN in respective polymorphs, different pairs of cutoff distances were chosen for the (2‐body, 3‐body) interactions: (4.5, 3.2) Å and (5.1, 3.6) Å were chosen for wurtzite (wurtzite) and rocksalt (rs) structures, respectively. We only considered these two structures since wurtzite‐AlN, rs‐GdN, and rs‐ScN are their respective ground‐state structures. To sample the composition space of the quaternary phase diagram, we considered an equally spaced composition grid with an increment of 0.083. In total, we have 91 grid points, including the binary compounds at the corners (AlN, ScN, and GdN).

Building on the DFT total energies of these grid points, we used the following regular solution model to model the mixing enthalpy for ${\mathrm{Al}}_{1-x-y}{\mathrm{Sc}}_{x}{\mathrm{Gd}}_{y}N$ alloys (x+y+z = 1),

(2) $$\Delta H_{mix}\left(x,y,z\right)=ax+by+cz+dxy+exy+fyz+gxyz$$

where a‐g are fitting parameters. d‐f and g are the interaction energies for two‐body and three‐body interactions, respectively.

Along with ideal mixing for entropy, $\Delta S_{mix}\left(x,y,z\right)=-k_{B}\left[xln\left(x\right)+yln\left(y\right)+zln\left(z\right)\right]$, we acquired an analytical equations for Gibbs mixing energy, $\Delta G_{mix}\left(x,y,z,T\right)=\Delta H_{mix(x,y,z)}-T\Delta S_{mix}\left(x,y,z\right)$, which can be used to predict the phase diagram of AlN–ScN–GdN. $k_{B}$ is the Boltzmann constant. To predict whether spinodal decomposition happens at a given composition and temperature, we followed the generalized approach for multicomponent systems [25, 26] to check if there is any negative eigenvalues of its $B(\bar{x}$,T) matrix,

(3) $$B\left(\bar{x},T\right)=P\frac{\delta^{2}}{\delta {\bar{x}}^{2}}\Delta G\left(\bar{x},T\right)$$

where $\bar{x}$ is the composition vector, $\bar{x}=\left(x,y,z\right)$. P is the projection matrix to ensure x+y+z=1 and has the following form, [25]

(4) $$P=I-J/m=\left[\begin{array}{ccc} 2/3 & -1/3 & -1/3\\ -1/3 & 2/3 & -1/3\\ -1/3 & -1/3 & 2/3 \end{array}\right]$$

where I and J are the m×m identity and ones matrices, respectively. m is the dimension and was set to three for a pseudo‐ternary (i.e., quaternary) alloy.

Using the analytical equation for $\Delta G_{mix}\left(\bar{x},T\right)$, we can evaluate its second derivative with respect to $\bar{x}$,

(5) $$\frac{\delta^{2}}{\delta {\bar{x}}^{2}}\Delta G\left(\bar{x},T\right)=\left[\begin{array}{ccc} \frac{k_{B}T}{x} & d+gz & f+gy\\ d+gz & \frac{k_{B}T}{y} & e+gx\\ f+gy & e+gx & \frac{k_{B}T}{z} \end{array}\right]$$

Any negative eigenvalues in $B(\bar{x}$,T) matrix indicate that the ${\mathrm{Al}}_{1-x-y}{\mathrm{Sc}}_{x}{\mathrm{Gd}}_{y}N$ alloys at the given composition and temperature will undergo spinodal decomposition.

In addition to identifying metastable (kinetically stable) regions, we also estimated the stable region using the Gibbs mixing energy. For a given effective temperature, we use the numerical approach of convex hull analysis to identify compositions that are on the hull with a tolerance of 10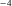 eV atom^−1^. The compositions make up the stable region. For all the reported phase diagram, a finer composition grid with the increment of 0.005 (20301 points in total) was used. The critical composition is temperature independent and was defined by the composition at which the mixing enthalpies of the competing phases were the same.

#### 4.2.3 Response to Electric Field

We used the finite difference approach implemented in VASP to calculate the self‐consistent response of ${\mathrm{Al}}_{1-x-y}{\mathrm{Sc}}_{x}{\mathrm{Gd}}_{y}N$ alloys to electric field. Electric field vector ($E_{x}$, $E_{y}$, $E_{z}$) = (0.01, 0.01, 0.01) eV Å^−1^ was used with fourth order finite difference stencil. Instead of SQS structures, we approximately sampled the statistics of cation disorders in wurtzite ${\mathrm{Al}}_{1-x-y}{\mathrm{Sc}}_{x}{\mathrm{Gd}}_{y}N$ alloys using a 72‐atom supercell. Each composition has four different structures with cations randomly distributed. A Γ‐centered Monkhorst‐Pack 4×4×2 ***k*** grid and stricter self‐consistency cutoff energy of 10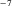 eV are used to ensure converged results. For ionic contributions to piezoelectric coefficient and elastic constant tensors, ionic displacement of 0.01 Å and the 2nd‐order central difference approach was used. VASP calculates the piezoelectric strain coefficients ($e_{ij}$) directly and were converted to the piezoelectric stress coefficients ($d_{ij}$) using the corresponding elastic constant tensor.

#### 4.2.4 Structural Distortion Factor

To quantify structural distortions in wurtzite AlN‐based alloys, we calculated the deviations in Al‐N bond lengths and N–Al–N bond angles from those in pure wurtzite AlN, based on DFT fully‐relaxed structures. We first identified all the Al‐N bonds with the cutoff radius of 1.15 times the shortest Al‐N. All the bonds were then grouped into basal and polar Al‐N bonds by the criterion of angles from wurtzite polar direction, (0,0,1), being less than 25.8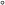. Reference basal and polar Al‐N bonds are 1.9014 and 1.9136 Å, respectively. Since alloying changes the volume, we scaled the reference Al‐N bond lengths by ${\left(\frac{V^{\prime }}{V_{0}}\right)}^{1/3}$, where $V^{\prime }$ and $V_{0}$ are the volumes of alloys and pure AlN, respectively, to accounted for the effect. The reference N‐Al‐N angles are 110.7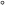 for angles between polar and basal Al‐N bonds. The reference angle is 108.2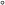 when only basal Al‐N bonds are considered. We considered all the Al‐N bonds and N‐Al‐N angles and calculated the difference from the referenced values.

Next, we devised a structural distortion factor using both the distributions of deviation in bond length and angles. We first normalized the distribution such that the sum of the distribution is one. We further normalized the deviations by the average Al‐N bond length (1.9043 Å) and angle (109.46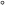), respectively. This also makes the structural distortion factor unitless. The distribution was then analyzed with a bin size of 0.01. Lastly, we integrated both distributions, $f_{\alpha }\left(x_{i}\right)$, using the following equation,

(6) $$I_{\alpha }=\sum_{i}\left|x_{i}\right|f_{\alpha }\left(x_{i}\right)$$

where α indicates the distribution of bond length or angle, and i runs over all the bins of the histograms. Lastly, structural distortion factor is the sum of the two.

#### 4.2.5 Electronic BandGap

We used the hybrid functional (HSE06) [70] to fully relax the 72‐atom supercells for ${\mathrm{Al}}_{1-x-y}{\mathrm{Sc}}_{x}{\mathrm{Gd}}_{y}N$ alloys and calculate their electronic band structure. The Brillouin zone was sampled using a Γ‐centered Monkhorst‐Pack 2×2×2 ***k*** grid with slightly larger cutoff kinetic energy of 400 eV. Bandgaps for a given composition were obtained by averaging over four different structures with randomly distributed cations.

#### 4.2.6 Polarization Switching Barrier

Polarization switching barriers were calculated using the solid‐state nudged elastic band (SS‐NEB) method to find the minimum energy path (MEP) between the positive‐ and negative‐polar structures of ${\mathrm{Al}}_{1-x-y}{\mathrm{Sc}}_{x}{\mathrm{Gd}}_{y}N$. [71] These calculations were carried out with the VASP Transition State Theory (VTST) tools as implemented in the vtst‐182 code developed by Henkelman et al. [72] The largest energy density difference between a peak and its adjacent valleys along the MEP was considered as the polarization switching barrier [50, 73]. Both positive‐to‐negative and negative‐to‐positive switching directions were considered. The initial intermediate paths were constructed via linear interpolation between the positive‐ and negative‐polar structures for all compositions and no further constraints were applied during SS‐NEB simulations.

#### 4.2.7 Spontaneous Polarization

The modern theory of polarization was used to calculate the spontaneous polarization, utilizing the Berry phase approximation to determine polarization in periodic systems accurately [74, 75]. We used the VASP 5.4.4 implementation, and (0.25, 0.25, 0.25) were chosen as the reference frame for dipole calculations. After the polarization switching MEPs are determined, algorithms implemented in Pymatgen were used to find smooth polarization paths to calculate the polarization quanta [76]. We visually confirmed the smoothness of the polarization pathway, and if the algorithm fails to determine a smooth path, we manually identified smooth polarization pathways [76].

## Conflicts of Interest

The authors declare no conflicts of interest.

## Supporting information


**Supporting File**: advs74057‐sup‐0001‐SuppMat.pdf.

## Data Availability

The data that support the findings of this study are available from the corresponding author upon reasonable request.
