# Supplementary material for: Phase Diagrams and Piezoelectric Properties of Wurtzite Al1−x−y Sc x Gd y N Heterostructural Alloys
Source: Adv Sci (Weinh). 2026 Jan 30:e20641. Online ahead of print. doi: 10.1002/advs.202520641 (PMC13326054; doi:10.1002/advs.202520641)
Supplement: Supplementary file 1 — Supporting File: advs74057‐sup‐0001‐SuppMat.pdf. [file ADVS-9999-e20641-s001.pdf]

— Supplementary Material —
Phase diagrams and Piezoelectric Properties of Wurtzite $Al_{1-x-y}Sc_xGd_yN$ Heterostructural Alloys

Julia Martin,[1] Cheng-Wei Lee,[2] Nate S.P. Bernstein,[2,1] Thi Nguyen,[3]
Ande Bryan,[2] Eli Cooper,[2] Sage R. Bauers,[1] Andriy Zakutayev,[1]
Keisuke Yazawa,[1,2,*] Prashun Gorai,[3,1,†] and Rebecca W. Smaha[1,2,‡]

[1]*National Renewable Energy Laboratory, Golden, Colorado 80401, USA*[§]
[2]*Colorado School of Mines, Golden, Colorado 80401, USA*[§]
[3]*Rensselaer Polytechnic Institute, Troy, NY 12180, USA*

CONTENTS

I. Additional Computational Data 2

II. Additional Composition Data 3

III. Additional XRD Data 4

IV. Additional Property Data 9

References 10

[*] Keisuke.Yazawa@nrel.gov
[†] goraip@rpi.edu
[‡] Rebecca.Smaha@NREL.gov
[§] Equal Contribution

I. ADDITIONAL COMPUTATIONAL DATA

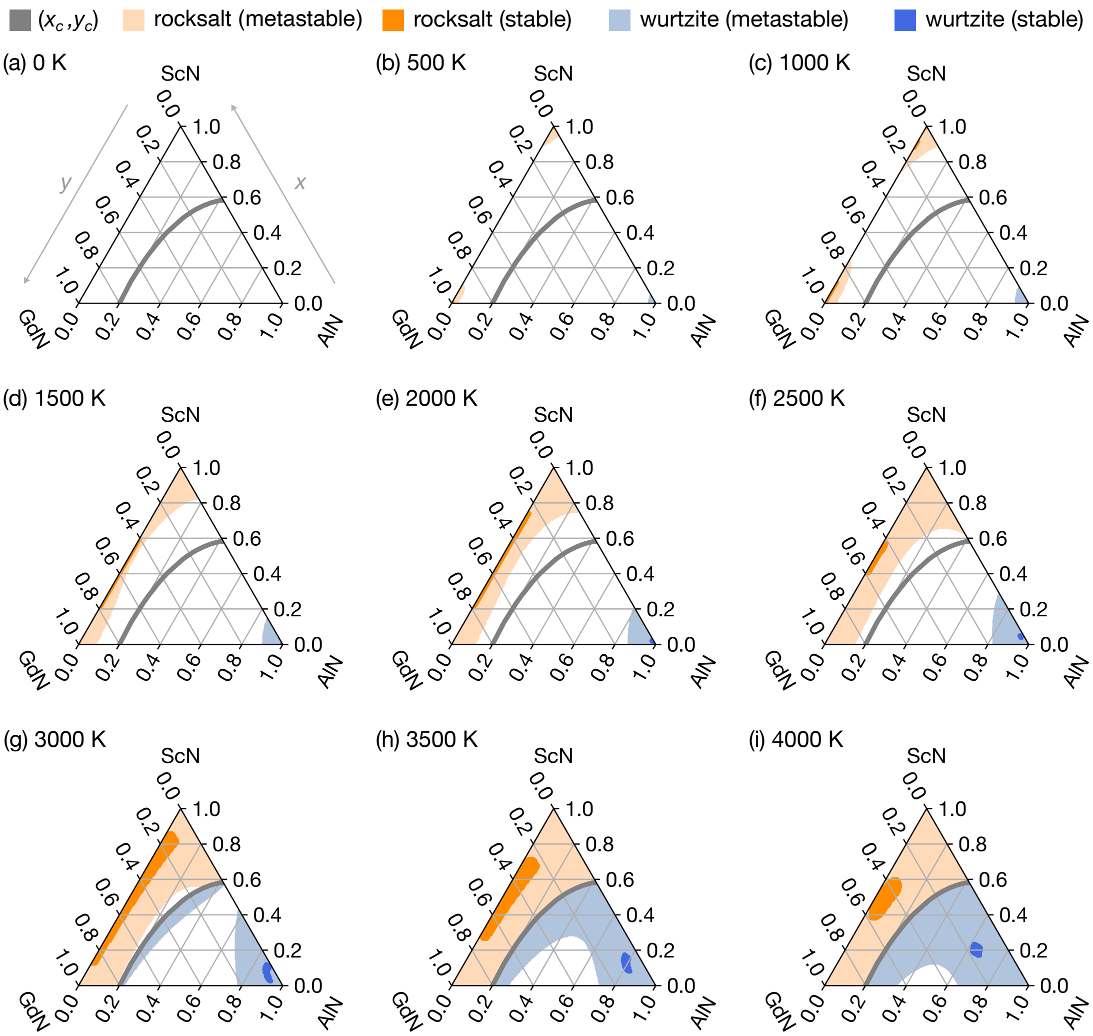

Figure S1. Calculated phase diagrams for $Al_{1-x-y}Sc_xGd_yN$ alloys across a range of effective temperatures (0 – 4000 K). The unstable regions are unshaded, and the metastable and stable regions are shaded.

II. ADDITIONAL COMPOSITION DATA

Table S1. Composition and anion atomic fractions for five representative $Al_{1-x-y}Sc_xGd_yN$ thin films as measured by EPMA.

Combinatorial Library Sample ID	Analysis Region of Sample Library Row	Al At.%	Sc At.%	Gd At.%	N At.%	O At.%	$\frac{(N)}{(N+O)}$	$\frac{(Al+Sc+Gd)}{(N+O)}$
C10_605_R3	Left	34.78	11.19	0.36	45.71	7.97	85.15	86.31
	Middle	35.73	11.09	0.24	45.96	6.98	86.82	88.89
	Right	35.80	10.90	0.19	47.27	5.83	89.02	88.31
C10_604_R3	Left	31.32	10.23	3.08	45.81	9.56	82.73	80.60
	Middle	32.40	10.13	2.30	45.64	9.53	82.73	81.26
	Right	33.76	10.13	1.50	47.26	7.35	86.54	83.12
C10_607_R3	Left	26.72	13.79	4.25	48.58	6.66	87.94	81.03
	Middle	28.28	13.90	2.99	48.89	5.94	89.17	82.38
	Right	29.81	13.95	2.14	49.72	4.40	91.87	84.77
C10_534_R4	Left	24.94	9.19	14.04	44.67	7.16	86.19	92.94
	Middle	28.23	10.02	10.43	45.68	5.64	89.10	94.86
	Right	29.57	9.94	7.27	47.70	5.52	89.63	87.90
C10_533_R4	Left	18.18	6.90	21.77	39.56	13.58	74.44	88.16
	Middle	22.27	7.97	16.76	43.09	9.91	81.30	88.68
	Right	25.80	8.96	13.32	45.08	6.84	86.83	92.60

III. ADDITIONAL XRD DATA

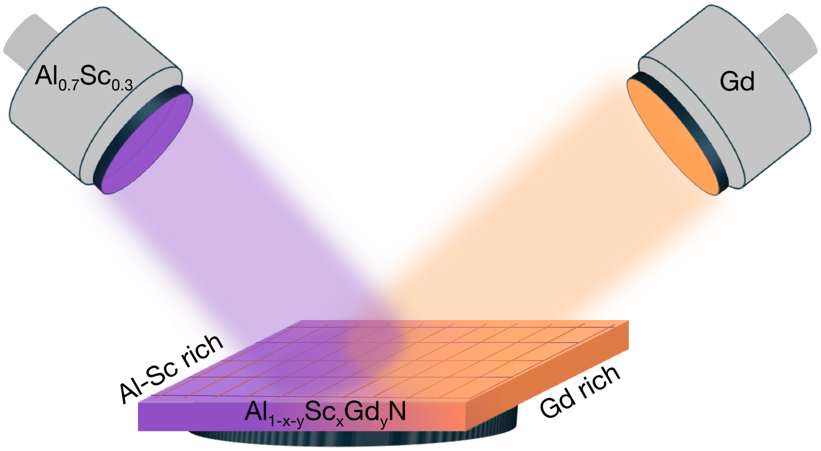

Figure S2. The geometry of the magnetron sputter chamber equipped with 3 RF guns that are positioned 120 degrees from each other. The resulting films demonstrate a composition gradient across the rows, yielding AlSc-rich and Gd-rich edges.

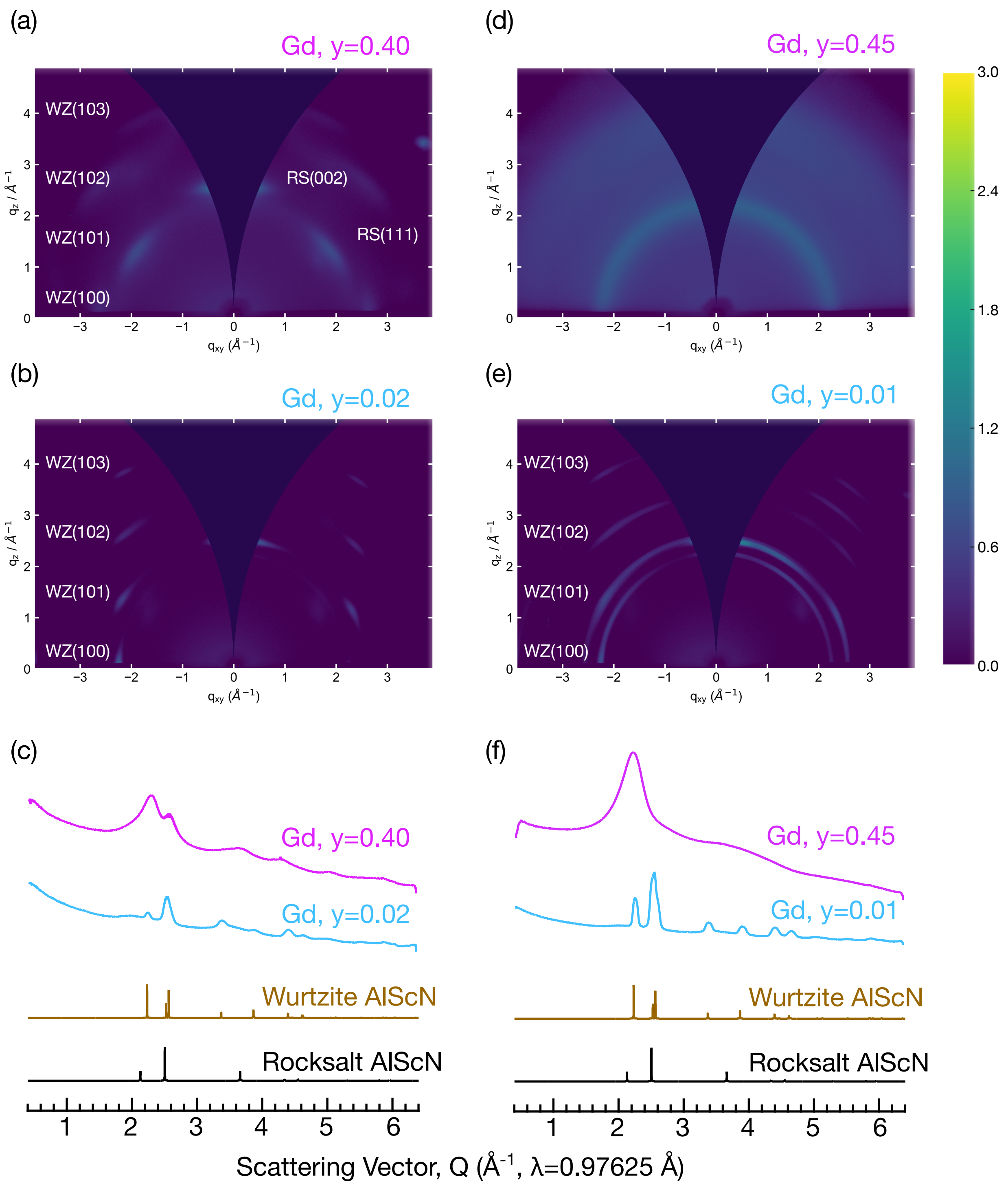

Figure S3. GIWAXS data for $Al_{1-x-y}Sc_xGd_yN$ grown under (a–c) N_2-rich conditions and d–(f) Ar-rich conditions. Panels (a) and (b) contain 2D detector images for the corresponding integrated data in (c), and panels (d) and (e) correspond to the integrated data in (f). For N_2-rich growths, low Gd substitution as in (b) generally yields phase-pure, well-textured wurtzite material, while higher Gd substitution yields a coexistence of wurtzite and rocksalt phases where the rocksalt phase dominates above y$\approx$ 0.30, as in (a). For Ar-rich growths, low Gd substitution as in (e) yields phase-pure but randomly oriented wurtzite $Al_{1-x-y}Sc_xGd_yN$ with an onset of amorphization at y$\approx$ 0.21 as displayed in panel (d).

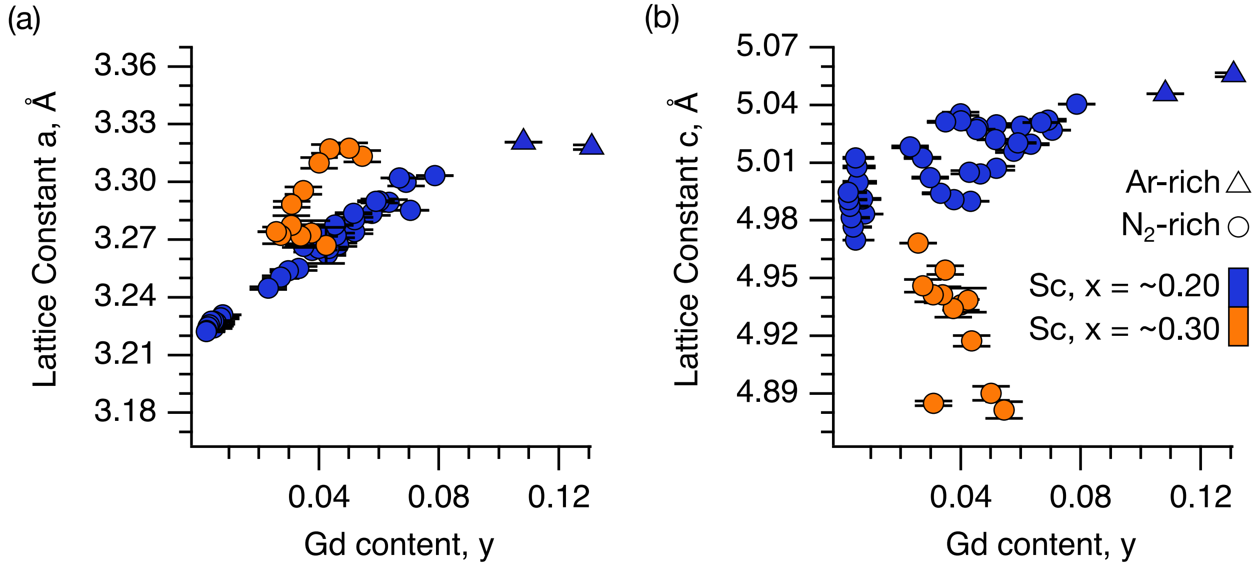

Figure S4. (a) Experimental lattice constant a as a function of Gd content for both $x \approx 0.20$ and $x \approx 0.30$. For Sc content $x \approx 0.20$, the lattice constant value does seem to increase linearly with increasing Gd content. A similar trend is seen in (b) for lattice constant c as a function of Gd content. In the case of x $\approx$ 0.30, a larger data set with higher Gd substitution is necessary to make any strong correlations between the lattice constants and Gd incorporation.

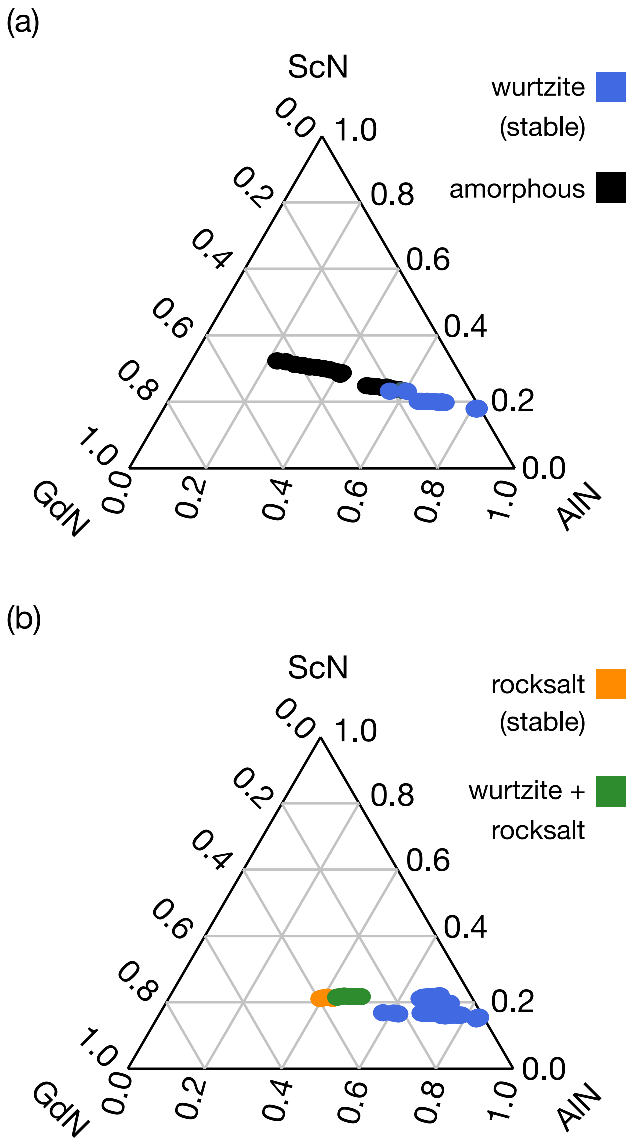

Figure S5. Experimental ternary phase diagram for $Al_{1-x-y}Sc_xGd_yN$ films grown under a) Ar- and b) N_2-rich chamber conditions.

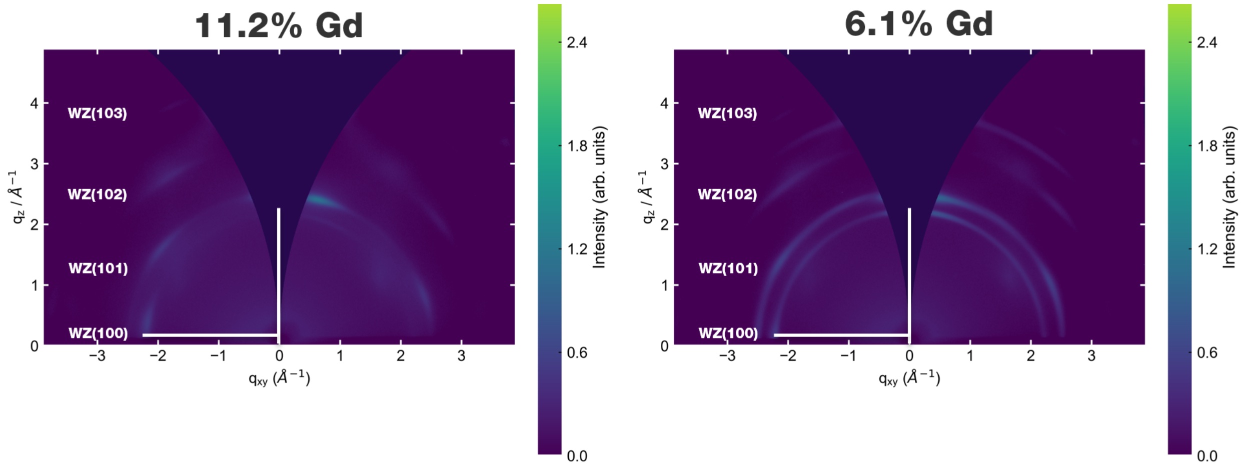

Figure S6. Synchrotron GIWAXS detector images of two points along the combinatorial row with Sc $x = 0.20$ upon which additional DBLI data were measured to extract $|d_{33,f}| = 1$ to 2 pm V^{-1}. Both 6 at.% and 11 at.% points on the combinatorial row show polycrystallinity, giving a possible explanation for the low values of $|d_{33,f}|$ observed. Both $y = 0.06$ and 0.11 images also show a slight indication of a rocksalt (111) peak at $Q_{xy} = 1.8$ and $Q_z = 1.1$

IV. ADDITIONAL PROPERTY DATA

The enhanced absorption at low energy seen in Fig. S7(b) for Sc content of $x = 0.3$ and Gd content of $0.05 \lesssim y \lesssim 0.12$ could be due to several causes: secondary phases with smaller band gaps, amorphous content causing broadening of α (Urbach tail), deep defects causing intra-gap states, or fitting artifacts.[1, 2] We note that similar behavior was observed in $Al_{1-x}Tb_xN$ at higher Tb incorporation, concurrent with the observation of a rocksalt phase, which should have a much lower bandgap.[3] It is therefore interesting that this low-energy absorption is observed at relatively low Gd incorporation at which GIWAXS data did not show the presence of a rocksalt phase, while the y≈0.20 sample did show rocksalt content but no low-energy absorption. As all samples that exhibit the low-energy absorption are from one combinatorial library, we attribute this behavior to defects intrinsic to that library. Nevertheless, the overall trend is the decrease in absorption onset (proportional to band gap) with increasing Gd and Sc substitution.

Additional DBLI data were collected for films containing 0.06 ¡ y ¡ 0.11 (Gd) and 0.2 ¡ x ¡ 0.23 (Sc), with detector images shown in Fig. S6. Values of $|d_{33,f}|$ were measured to be between 1 and 2 pm/V. This is the opposite trend of what would be expected for increased Gd content. An increase in $|d_{33,f}|$ is expected to occur for increasing Gd content based on the same lattice softening argument previously stated and based on the predictions in Fig. **??**. Plausible explanations for the decreased $|d_{33,f}|$ are: (1) a lack of texture, (2) the presence of a rocksalt phase, or (3) a mixed growth polarity of both nitrogen-polar and metal-polar orientations. Figure S6 shows a lack of texture for the Gd $y = 0.06$ and 0.11 points on the combinatorial sample with Sc $x = 0.20$. Both $y = 0.06$ and 0.11 plots show a slight indication of a rocksalt (111) peak at $Q_{xy} = 1.8$ and $Q_z = 1.1$, implying a possible rocksalt phase present throughout the film. Rocksalt is a centrosymmetric structure and therefore would reduce the effective piezoelectric coefficient if present. Both the lack of texture and the possible presence of rocksalt are consistent with the experimental decrease in $|d_{33,f}|$ and may be improved with future optimization.

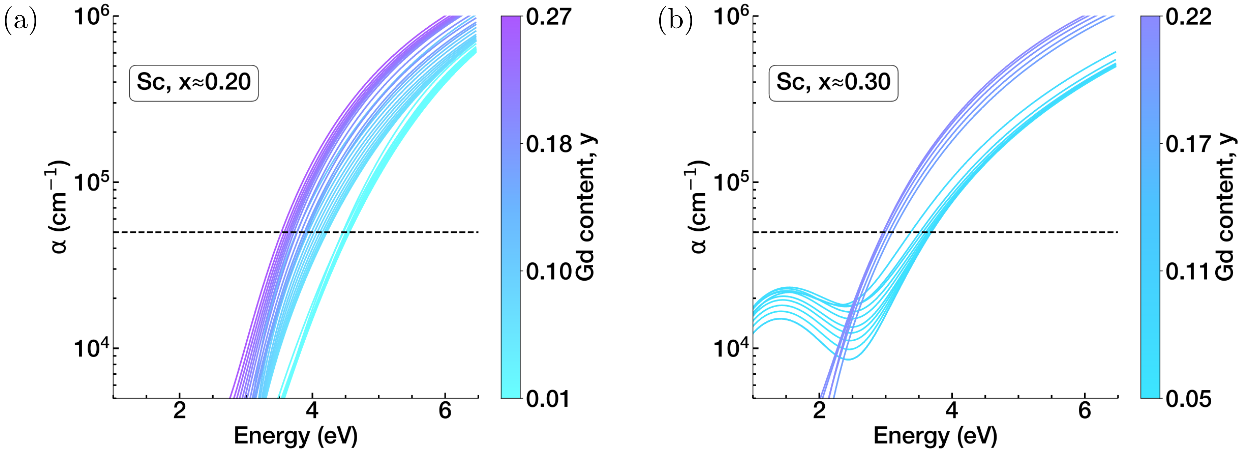

Figure S7. Ellipsometry measurements show the material system absorption onset decreases with increasing Gd concentration, y. Optical absorption coefficient (α) vs. energy are shown for (a) Sc content of $x \approx 0.2$ and (b) Sc content of $x \approx 0.3$. Both (a) and (b) show a dashed line indicating the energy of absorption onset value chosen as $\alpha = 5 \times 10^4$ cm^{-1} ($E_{\alpha=5\times10^4}$).

[1] S. Aljishi, J. D. Cohen, S. Jin, and L. Ley, Band tails in hydrogenated amorphous silicon and silicon-germanium alloys, Phys. Rev. Lett. **64**, 2811 (1990).
[2] D. V. Likhachev, N. Malkova, and L. Poslavsky, Quantitative characterization and modeling of sub-bandgap absorption features in thin oxide films from spectroscopic ellipsometry data, AIMS Materials Science **2**, 356 (2015).
[3] B. Paudel, J. S. Mangum, C. L. Rom, K. Egbo, C.-W. Lee, H. Guthrey, S. Allen, N. M. Haegel, K. Yazawa, G. L. Brennecka, and R. W. Smaha, Combinatorial synthesis and characterization of thin film $Al_{1\text{-}x}RE_xN$ (RE = Pr^{3+} and Tb^{3+}) heterostructural alloys, Journal of Materials Chemistry C **12**, 19620 (2024).
